# A Living Ethics Project to Address Psychological Distress in Chronic Illness: Process and Outcomes

**DOI:** 10.1111/hex.70457

**Published:** 2025-12-17

**Authors:** Bénédicte D'Anjou, Katherine Desjardins, Julie Ianniruberto, Danielle Méthot, Valérie Poulin, Rémi Rabasa‐Lhoret, Eric Racine

**Affiliations:** ^1^ Pragmatic Health Ethics Research Unit Institut de recherches cliniques de Montréal Montréal Québec Canada; ^2^ Institut de recherches cliniques de Montréal Montréal Québec Canada; ^3^ Institut de recherches cliniques de Montréal Université de Montréal, Centre Hospitalier de l'Université de Montréal Montréal Québec Canada; ^4^ Pragmatic Health Ethics Research Unit, Institut de recherches cliniques de Montréal Université de Montréal; McGill University Montréal Québec Canada

**Keywords:** chronic disease, complex disease, living ethics, living lab, psychological distress, rare disease

## Abstract

**Introduction:**

Individuals living with a complex or rare chronic disease live a life where the use of healthcare services and self‐care are part of their quotidian and even of their identity. They may, as a result, experience significant psychological distress. Yet, specialized healthcare providers (HCPs) who manage their care are often ill‐equipped to respond to the emotional or social dimensions of their patients' illness and intervene in a meaningful way.

**Methods:**

In this paper, we report on the process and outcomes of a living ethics project, structured as a living lab. The living lab followed a five‐phase methodology, each phase involving various research methods (e.g., semi‐structured interviews, group interviews) and oriented toward distinct tasks: identifying the issue (phase 1: problem identification), deepening understanding (phase 2: problematization), co‐developing interventions (phase 3: ideation), implementing them (phase 4: enactment), and evaluating the interventions and the overall process (phase 5: evaluation).

**Results:**

Phase 1 led to the identification of neglected psychological distress of patients as an important ethical issue. Phase 2 exhibited causes and consequences of psychological distress. Phase 3 led to the co‐development of: (1) an electronic medical appointment preparation form for patients, aimed at guiding medical consultations based on their specific needs, facilitating communication, and opening discussions about mental health; (2) a directory of mental health resources intended for clinic staff to better equip them in addressing the mental health of patients; and (3) mental health awareness posters with catchy slogans strategically placed throughout the clinic to raise awareness about mental health and encourage open discussions. Phase 4 led to the implementation of these interventions and phase 5 to their evaluation. All interventions were evaluated positively as well as the participatory nature of the research project while many core aspects of living ethics were furthered.

**Conclusion:**

This project shows that directly engaging stakeholders in ethics research, by addressing the moral issues they deem significant and working with them to tackle those issues rather than conducting research on them, can lead to tangible, unexpected, and positive moral and clinical outcomes, even within a short timeframe and with limited resources.

## 1 Patient or Public Contribution

Patients and clinic staff where the project was conducted were actively involved in all phases and aspects of the study. A project team made up of key stakeholders from the clinic supported the initiative throughout its development. In this role, they contributed to shaping the study design, including decisions regarding participant recruitment, interview questions, and strategies for collaborating with stakeholders. They also took part in the preliminary analysis and interpretation of the data after each phase, providing feedback that led the research team to revisit and refine the analysis before presenting the results to stakeholders. In addition, the project team carefully reviewed and commented on this manuscript. Their feedback was incorporated by the research team to strengthen the final version. Participating patients and all clinic staff were also meaningfully involved in all phases of the project, notably in determining the subject of the study, contributing to how the issue was understood, suggesting and co‐developing the interventions, and evaluating the interventions and the project.

## 2 Introduction

Individuals living with a complex or rare chronic disease live a life where the use of healthcare services and self‐care are part of their quotidian and even of their identity [1]. The ongoing routine of monitoring blood glucose and injecting insulin for Type 1 diabetes or of taking extreme sanitary precautions for people living with rare primary immunodeficiencies [2] can generate fatigue and psychological distress [3, 4]. Healthcare providers (HCPs) who offer care to these patients are often highly specialized and focused on treating these complex physical conditions. They are not always trained, equipped, or supported to respond to the emotional or social dimensions of their patients' illness ([4] even tough recent guidelines have tended to include the need for mental health support (e.g., Canadian diabetes guidelines [5]). Additionally, in contexts like Canadian public healthcare systems where we conducted our work, some major difficulties in accessing primary and mental healthcare [4, 6, 7] can leave many patients with chronic illnesses without the integrated care they need. This creates morally problematic situations where specialized HCPs, under constant pressure to see more patients within tight schedules, are confronted with the psychological distress of their patients and lack the means to intervene in a meaningful way. Patients, in turn, may endure a double burden: psychological distress caused by their health condition and additional suffering due to the lack of adequate support. This calls for the creation of ethical spaces where difficult situations experienced by patients and HCPs such as this can be voiced and tackled [8]. Creating such spaces is also one of the chief purposes of work oriented by a living ethics stance [9, 10] which supports the use of participatory research approaches such as living labs to address real world issues in specific clinical environments.

Participatory action research approaches offer a way to meet this need by engaging stakeholders to identify a common issue and then collectively envision, create, and evaluate potential responses. We know of no other initiatives (e.g., living lab, participatory study) on health ethics issues in the context of rare and chronic illness especially on the neglect of psychological distress as an ethical issue. However, there is related relevant literature. Participatory action research—which differs but has strong affinities with living labs [11]—has been long used to engage stakeholders in the context of mental health research (e.g., research on suicide, stigma, mental health care and delivery [12]. Participatory research has also been used to improve technology and care in “physical” chronic illness (e.g., cancer, diabetes) often with the goal of developing healthcare interventions and technology that better respond to patient expectations [13, 14, 15]. There is also a small participatory research literature on psychological distress in “physical” chronic illness and rare diseases. This literature is particularly pertinent to our study and helps support its relevance. For example, participatory action research studies in the perinatal context have attempted to produce interventions to reduce postnatal distress [16] and address postpartum depression in Indigenous women [17]. Cancer is certainly an area where psychological distress occurs, and participatory research has contributed to the development of new clinical interventions such as a group intervention [18] and adaptations to validated psychological interventions [19]. The intensive care unit (ICU) is also an area where work—conducted in parallel by our group in the form of another living ethics lab—has tackled the moral distress associated with inappropriate levels of care [20, 21]. Finally, another noteworthy area concerns psychological distress related to the COVID‐19 pandemic where participatory action research studies have supported the development of tailored interventions to promote relationships [22] and the gathering of more reliable and quantitative data given the ability of this kind of research promote partnerships and engagement [23].

Within participatory research approaches, living lab methodology is a promising route. Living labs bring research activities into natural environments, to address everyday problems with the stakeholders who experience them and support the trialing of new innovative solutions to these problems, often in the form of pilot interventions and prototypes [24, 25]. Living labs are thus sometimes described as ecosystems because of this naturalistic orientation and because of their goal of generating innovation from and through lived experiences [26]. They are notably consistent with the principles and orientation of a living ethics stance [9, 10], that is, a new orientation co‐developed by scholars, clinical ethicists, patients, and HCPs that attempts to bring ethics closer to everyday life in contrast to traditional top‐down and regulatory approaches often seen in ethics. Six methodological guideposts have been proposed to describe living ethics initiatives: (1) experientially grounded, (2) fostering flourishing, (3) supporting practices of co‐learning, (4) promoting dialogue and epistemic justice, (5) empowering and action‐oriented, and (6) co‐imagining futures [9]. Much like living labs, living ethics calls for a deep existential engagement with difficult experiences, while maintaining a clear focus on responding through action, collaboration, and co‐learning. Living labs and the living ethics stance thus appear to be promising orientations for creating ethical spaces where moral issues encountered in healthcare contexts can be identified and tackled.

In this paper, we report on the process and outcomes of a living ethics project, structured as a living lab, on the neglected psychological distress of individuals living with a complex or rare chronic disease—an issue identified through the project itself. This project, conducted in collaboration with patients and clinicians, consisted of five phases: (1) problem identification, (2) problematization, (3) ideation, (4) enactment, and (5) appreciation. Given the iterative nature of living labs where one phase of research prepares the other, this paper provides an integrated overview of the project's five phases to convey a coherent and continuous account of the study. Accordingly, the methods and results for each phase are presented together, akin to studies that report successive experiments sequentially. The initial phases describing the problem of neglected psychological distress yielded extensive data and have been reported elsewhere [4, 27]. Accordingly, this paper focuses on the interventions co‐developed, the project outcomes, and especially their evaluation by stakeholders, but we present a summary of the first two phases to help readers understand the globality of the project and its progression.

## 3 Methods and Results

**Figure 1:**
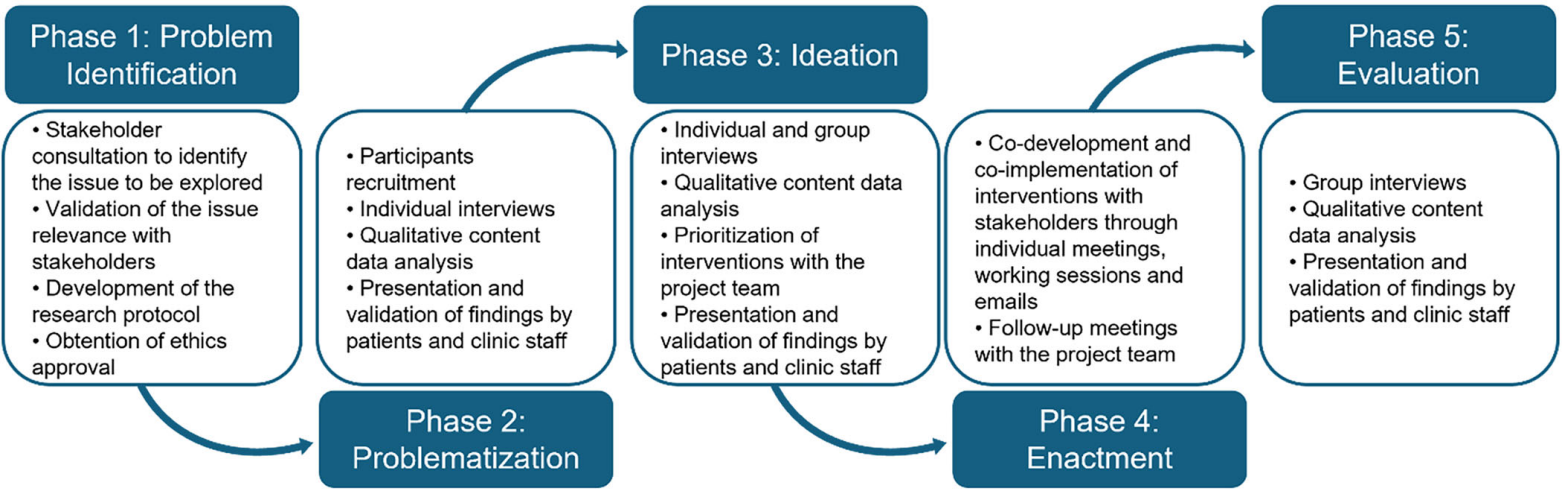
Living ethics lab process overview.

This living ethics project was conducted over a 24‐month period (from June 2022 to June 2024) in a highly specialized interdisciplinary care clinic located in Montreal (Quebec, Canada). Embedded within a publicly funded research center and affiliated with a university hospital, the clinic serves as a reference center for specialized medical care and clinical research in seven areas of care: cardiovascular prevention, genetic dyslipidemia, diabetes, hypertension, primary immunology, long COVID, and rare kidney diseases. The project was carried out in close collaboration with clinic patients and staff (i.e., administrative staff, doctors, nurses, genetic counsellors, kinesiologists, nutritionists, and research professionals). Each of the five phases of the living lab project involved various research methods (see Figure 1) and was oriented toward a distinct focus: identifying the issue (phase 1: problem identification), deepening understanding (phase 2: problematization), co‐developing interventions (phase 3: ideation), implementing them (phase 4: enactment), and evaluating the interventions and the overall process (phase 5: evaluation). The methods and findings for each phase are presented below.

### 3.1 Phase 1: Problem Identification

#### 3.1.1 Methods

As a form of participatory research, living labs in ethics should start open‐endedly to create room for stakeholders to identify morally significant issues that matter to them. Accordingly, in the initial phase of the project (June–December 2022), a project team comprised of key members of the clinic (the operations manager (K.D.), reception coordinator (J.I.), deputy director (D.M.) and clinic director/physician (R.L‐R.))^1^ was created to oversee the project and facilitate interactions with patients and clinic staff, i.e., the stakeholders in this project. Project team members were recruited based on their interest and willingness to contribute to the project and were supported by an executive team composed of research staff (i.e., a research director (E.R.), a research coordinator (B.D.), and research assistants). The executive team informally consulted patients (*n* = 3) and clinic staff (*n* = 8) individually to identify a moral problem to be addressed. A subsequent presentation to the entire clinic staff validated the relevance of the issue identified. A complete research protocol was then developed and submitted for ethics review (approval no. 2023‐1205 from the Research Ethics Board of the Montreal Clinical Research Institute (IRCM)).

#### 3.1.2 Results

The initial consultation process with patients and clinic staff led to the identification of the issue of psychological distress among people living with a rare or complex chronic disease—a common concern among consulted parties—as the study's focus (see previously published papers [4, 27] for more details). The management of psychological distress was felt to be suboptimal and neglected such that HCPs felt like they failed to deliver appropriate, quality, and ethical care to their patients because they were not able to respond to the needs and expectations of patients as whole people. For patients, not being offered support and resources, or even attentive listening, generated several problems such as feeling not being respected as a person, not being heard (being dismissed and invalidated), being unsupported, not being treated fairly, in ways that put on the line important moral values. These experiences of distress and their neglect did not necessarily occur in the clinical environment of the study at hand but patients brough this legacy to this clinical environment.

### 3.2 Phase 2: Problematization

#### 3.2.1 Methods

Once the issue was identified, there was a need for its in‐depth understanding. During the problematization phase (January–June 2023), 22 individuals living with a rare or complex chronic condition and clinic staff members took part in semi‐structured individual interviews. Recruitment, data collection and analysis methods are detailed in a previous publication [4].

#### 3.2.2 Results

Six broad topics intrinsically related to patients' psychological distress were identified and explored with and by participants: (1) the concept of psychological distress, (2) causes of patients' psychological distress, (3) consequences of patients' psychological distress, (4) managing patients' psychological distress within the healthcare framework, (5) mitigating factors of patients' psychological distress, and 6) potential avenues for improvement (see [4] for more details). Overall, psychological distress was found to be a substantive issue. For patients, it “exacerbated physical discomfort and disease symptoms, decreased attendance and responsiveness to treatment or follow‐up, self‐medication, and social isolation” [4]. For clinic staff: it led to “more complex clinical interactions, enhanced or deteriorated care relationships, and feelings of frustration, powerlessness, and guilt” [4].

### 3.3 Phase 3: Ideation

#### 3.3.1 Methods

Once patients' psychological distress was better understood, a concerted effort was made to co‐imagine potential solutions. During the ideation phase (July–September 2023), nine individual interviews and four group interviews were conducted with 23 participants (i.e., 14 patients and 9 clinic staff) to identify and elaborate on interventions aimed at mitigating psychological distress. Structured as working sessions, these recorded interviews explored the anticipated benefits, limitations, and implementation process of each proposed intervention. The data collected were then transcribed and coded through an iterative collaborative process involving the executive and project teams, supported by the software MAXQDA and inspired by the basic principles of qualitative content analysis [28]. Based on these results, interventions were classified according to their relevance to the problem at stake and feasibility in terms of the resources available. This then led to a prioritization exercise carried out by the executive and project teams. The prioritized interventions were then presented to and discussed with clinic staff and patients participating in the study via in‐person and online presentations, specifying each person's potential roles in the co‐development and co‐deployment of interventions.

#### 3.3.2 Results

A total of 17 potential interventions were suggested and considered by participants. Among these, nine were considered feasible and relevant, five were deemed less feasible, and three were considered less relevant (see Table 1). Three interventions were prioritized: (1) co‐develop a mental health resource directory aimed at clinic staff, (2) co‐develop a medical appointment preparation form for patients, and (3) co‐develop and display posters to promote discussions about mental health between patients and clinic staff (see Table 2).

**Table 1:** Potential avenues for improvement.

Feasible and relevant interventions (N = 9)
Develop a medical appointment preparation form for patients to help both patients and clinic staff better prepare for appointments. Provide mental health training sessions for clinic staff to better equip them. Develop a mental health resource directory to better equip clinic staff. Establish discussion/support groups for clinic patients. Develop a resource webpage for clinic patients. Organize scientific cafés* for patients and clinic staff. Develop a protocol for critical mental health situations to better equip clinic staff. Develop and display posters to promote discussions about mental health between patients and clinic staff. Establish a patient committee to better integrate patient perspectives into decision‐making. Develop mental health referral guidelines for clinic staff, helping them refer patients to appropriate mental health services and resources.
Less feasible interventions (N = 5)
Hire a mental health professional at the clinic. Establish a peer support program for clinic patients at the clinic. Redesign examination rooms to foster discussions about mental health between patients and clinic staff. Recruit psychology interns at the clinic to provide mental health support to patients. Organize recurrent team discussions on patients' mental health among clinic staff.
Less relevant interventions (N = 3)
Facilitate access to clinic parking. Organize and celebrate an annual Rare Disease Day at the clinic. Offer medical appointments remotely.

*Note:* Table 1 presents an overview of all interventions suggested by participants. These were classified based on their feasibility and relevance within the context of the project.

*Scientific cafés are forms of public discussions about research hosted in informal venues such as cafés and pubs [29]. They have been supported by Canadian institutions and funding agencies.

**Table 2:** Detailed analysis of prioritized interventions.

	(1) Mental health resource directory	(2) Medical appointment preparation form	(3) Posters promoting discussions about mental health
Description	A directory of mental health and other resources for patients and their relatives Containing general and specific resources on the various health conditions treated at the clinic Accessible to all clinic staff and updated regularly	Confidential, optional form including 3 or 4 questions on: patients' expectations regarding the appointment, their questions, the state of their mental health and their desire to discuss it during the appointment, significant health events experienced since the last appointment Open‐ended questions to guide reflection Sent 5–7 days before their medical appointment as an attachment to the appointment confirmation email Would be consulted by the medical team before the appointment Possibility of customizing the questionnaire for each clinic	Posters in exam rooms, doctors' offices and the waiting room with catchy slogans to promote and normalize discussions about mental health between patients and the healthcare team
Justification and anticipated benefits	Would allow clinic staff to become more familiar with available resources and be better equipped to handle sensitive mental health situations Could help improve patients' care experience Would support better communication and standardization of approaches Addresses a current need: since clinic staff are easily accessible, patients often reach out for support on various life issues for which clinic staff are not always aware of the appropriate resources	Would provide the medical team with a clearer understanding of the patient's condition, needs, expectations, concerns, and health follow‐up, allowing them to prepare accordingly Would help structure the appointment around the patient's needs and expectations Would reduce the pressure on patients to ask questions and lower the risk of them leaving with unanswered concerns Would open and facilitate conversations about psychological distress, enabling earlier intervention Would offer a more comprehensive overview of patients' overall health and improve information sharing Would allow for individual consultation rooms to be reserved for patients wishing to discuss mental health during their appointment	Would help normalize and open discussions on mental health
Co‐development and co‐deployment process	Survey each clinic's staff about resources known or used Document resources, their contact details, language, cost, means of contacting them, etc.	To develop the form, it would be necessary to consult: clinic patients, clinic staff, mental health specialists Before and during implementation, it would be important to introduce and promote the purpose of the form to patients and staff (e.g., by e‐mail and posters)	Consult stakeholders to develop catchy slogans Collaborate with the communications department
Potential challenges and limitations	Laborious work Need to update the directory regularly Difficulty in establishing a comprehensive and relevant directory of resources as they vary according to the area where patients reside Staff's lack of familiarity with resources can be a barrier to referring patients to them	The mental health section may trigger distress in some patients The clinical team may lack the time to consult the form before the appointment Some patients and staff members may be unwilling to engage with this new initiative Patients' mental health needs may exceed the clinic staff's expertise or scope of practice Successful implementation requires that clinic staff be familiar with mental health resources and feel comfortable discussing these topics	May go unnoticed or have limited impact on the issue at hand Requires that the clinical team have sufficient time, an open attitude, appropriate skills, and access to resources to discuss mental health effectively

*Note:* Table 2 presents the detailed analysis for the three interventions that were prioritized. This analysis includes their description, justification and anticipated benefits, co‐development and co‐deployment process, and potential challenges and limitations.

### 3.4 Phase 4: Enactment

#### 3.4.1 Methods

Once interventions were identified and prioritized, a separate process of developing and deploying them took place (September 2023–April 2024).

For the mental health resource directory, clinic staff were consulted in person and by e‐mail to share all relevant resources for patients and their families that they were aware of and used in their practice. Additional relevant resources were subsequently found through online searches conducted by the executive team. All identified resources were compiled along with their contact details, target population, and the region covered. These resources were then organized into two main types of categories: general support categories (coping with illness, eating disorders, rare diseases, patients' rights, and caregiver support) and categories aligned with the clinic's areas of care (diabetes, hypertension, immunology, lipid disorders, and rare kidney diseases). To make the directory available to clinic staff, an electronic version was added to the virtual staff resource file and emailed to all clinic staff at the official launch of the interventions. Hard copies were also printed and placed at the clinic reception desk.

The medical appointment preparation form was developed iteratively over five working sessions involving clinic staff and patient participants. During these sessions, participants selected and formulated the form's questions, and envisioned its practical implementation. The executive team notably ensured that patients had the final say in both the choice and wording of questions, as the form was specifically designed for their use. To facilitate its implementation, the executive team worked with the project team and information technology services to integrate the form into the electronic medical record and to send it to patients before their medical appointment. Finally, several strategies were deployed to encourage its use. First, an explanatory document for patients was created by the executive team and joined to the form. The project team also developed and shared a support document to help clinic staff access completed forms online. Additionally, the executive team met individually with a physician from each clinic to present the form, explain its functionality and anticipated benefits, and to discuss strategies for incorporating it into their practice to ensure that completed forms would be consulted by the clinical team before patient appointments. Finally, promotional posters were developed by the executive team with the help of the communications department.

Concerning the posters to promote discussions about mental health between patients and clinic staff, participants were consulted (during working sessions and meetings related to the development of the medical appointment preparation form) to suggest catchy slogans and give their opinion on the ones they preferred. In collaboration with the project team, two slogans were then selected. Finally, the executive team worked with the communications department to develop the posters.

These three interventions were officially launched through a presentation and an email sent to all clinic staff and patient participants, along with the display of the promotional medical appointment preparation form poster and posters promoting mental health discussions in strategic locations throughout the clinic (waiting room, examination rooms, and doctors' offices). Following the launch, the executive team oversaw the implementation of the interventions through monthly meetings and ongoing communication with the project team to monitor their adoption by clinic staff and patients, and to assess if any adjustments were needed.

#### 3.4.2 Results

The enactment phase ultimately resulted in the implementation of the three targeted interventions, and even prompted additional ones (as reported below).

**Figure 2:**
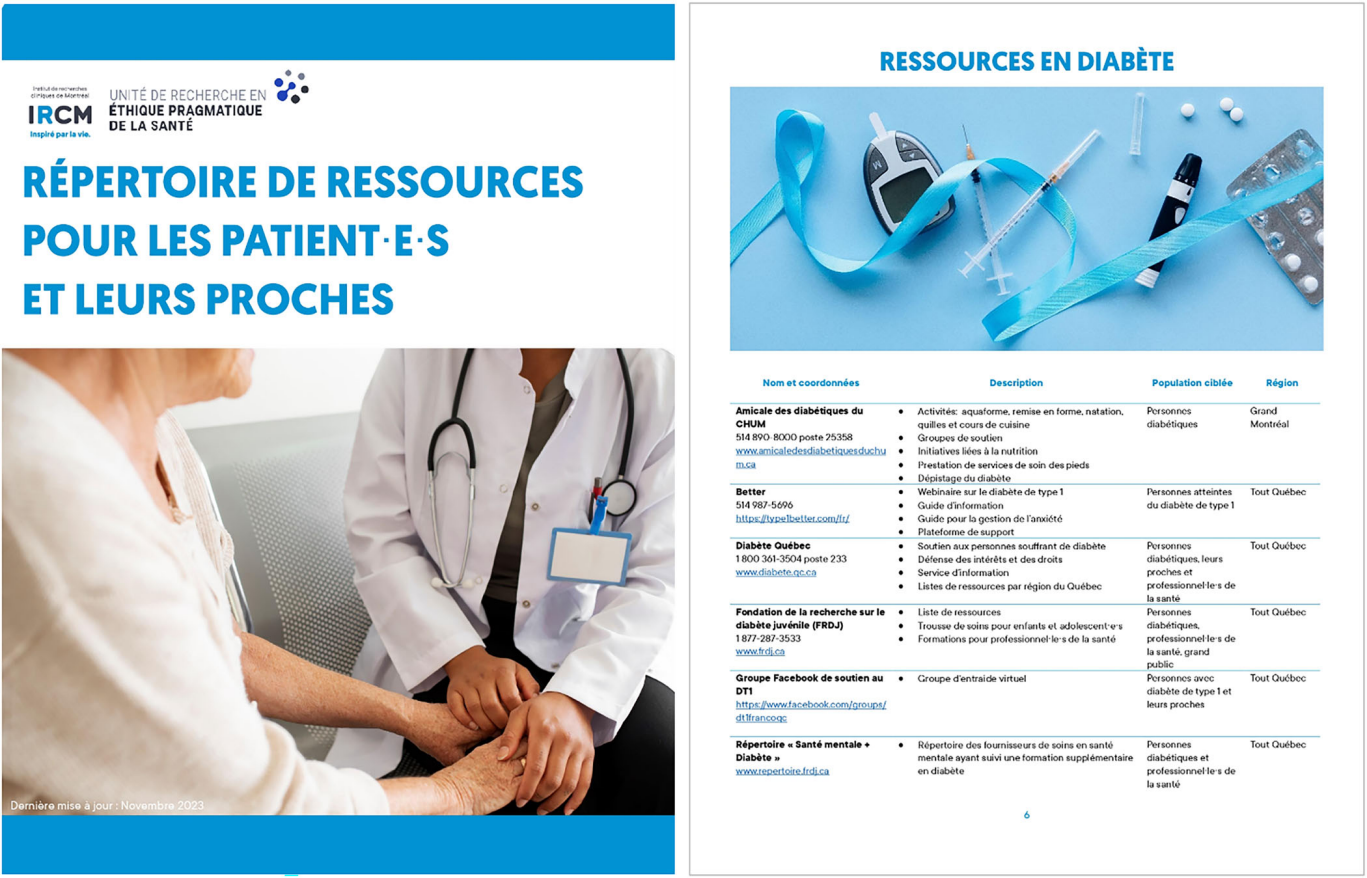
Mental health resource directory.

The first intervention was the co‐development of the resource directory (see Figure 2 and Supporting File S4), which was designed to provide clinic staff with additional resources to offer to patients and their families. This directory includes not only resources to better support patients experiencing psychological distress, but also a range of resources to help patients and their relatives navigate with their illness, thereby potentially preventing the onset of psychological distress.

**Figure 3:**
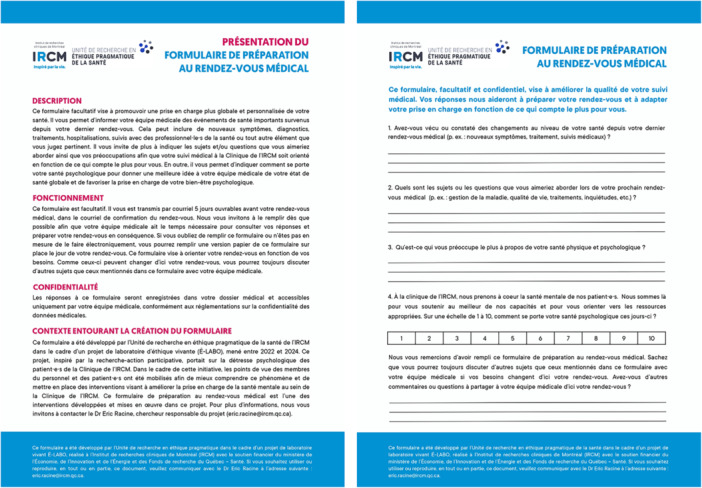
Electronic medical appointment preparation form.

The second intervention consisted in co‐developing a medical appointment preparation form for patients (see Figure 3 and Supporting File S1). This optional four‐question form was designed to foster a more holistic and personalized approach to patient care by allowing patients to inform their clinical team of significant health events that happened since their last medical appointment, indicate topics, questions, or concerns they wish to discuss, and self‐assess their psychological well‐being.

**Figure 4:**
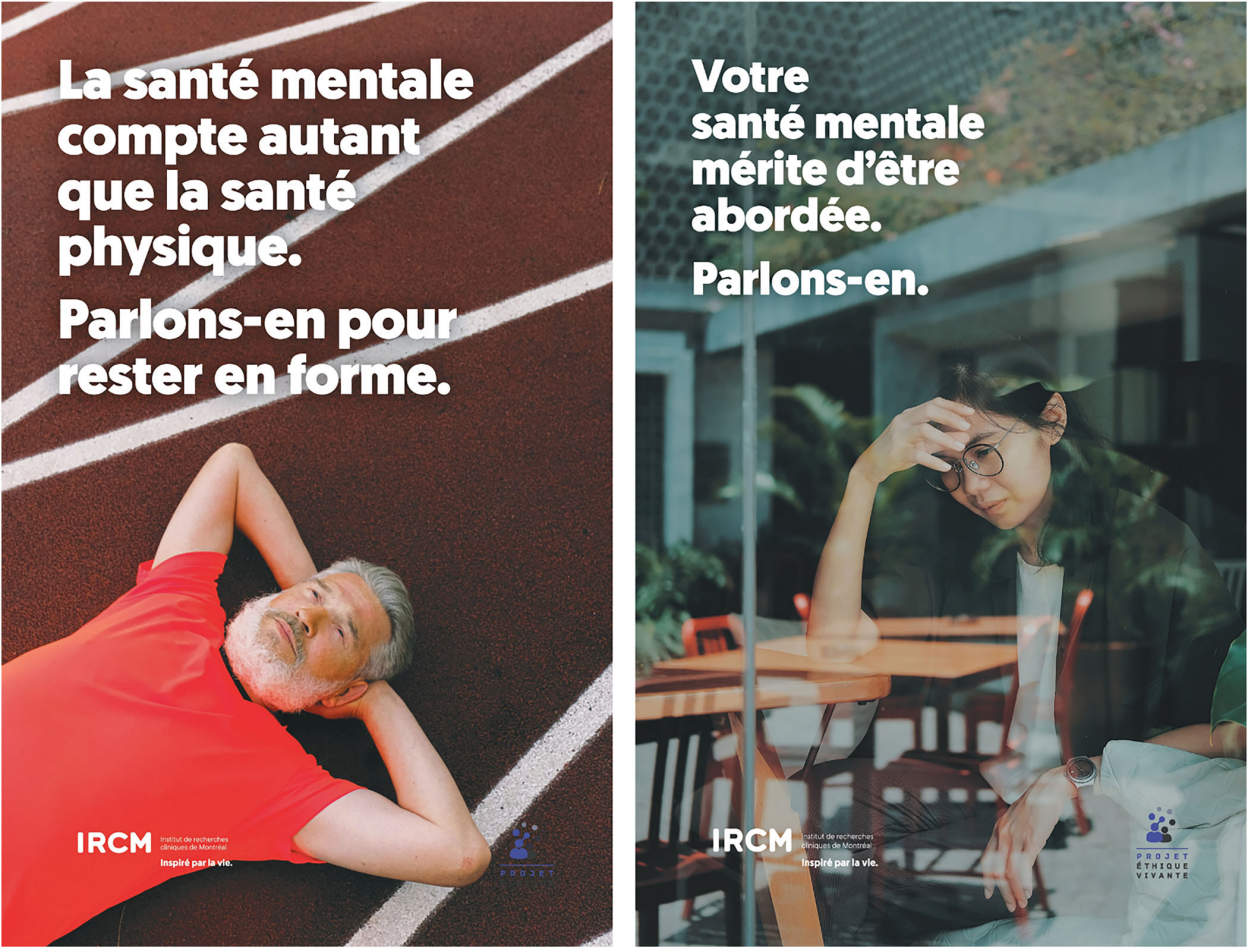
Mental health awareness posters.

The third intervention was the co‐development and display of posters promoting discussions on mental health between patients and clinic staff (see Figure 4 and Supporting Files S2 and S3).

In parallel with the interventions led by the executive team, the clinic's management team took charge and deployed additional interventions to address the issue. They provided suicide prevention training to clinic staff to better equip them with knowledge of existing resources and appropriate actions to take in such situations. Additionally, they developed mental health referral guidelines to help staff direct patients to appropriate mental health services and resources. Furthermore, the resources identified and compiled in the directory led them to create a patient resource web page on the clinic's website as well as a small card distributed to patients listing general mental health resources.^2^ These are all interventions that were suggested by participants within the framework project but they were implemented separately due to limited time and resources of the research team.

### 3.5 Phase 5: Appreciation

#### 3.5.1 Methods

Four months after the interventions were launched, an evaluation phase (April–June 2024) took place [30, 31] to understand the outcomes of the interventions and of the overall project. An interview grid aimed to obtain participants' perspectives on: (1) the interventions, (2) the participatory research process, (3) the outcomes of the project based on the methodological aspects of the living ethics stance (i.e., co‐learning, well‐being, co‐imagination, communication/dialogue, and empowerment [10]), and (4) the challenges and limitations of the project. Semi‐structured individual interviews, lasting between 15 and 60 min, were conducted with 17 participants (i.e., 8 patients and 9 clinic staff). The interviews were transcribed and coded through an iterative, collaborative process involving the executive and project teams, supported by MAXQDA software and inspired by the basic principles of qualitative content analysis [28]. Preliminary results were subsequently presented to clinic staff and patient participants via in‐person and virtual presentations to confirm if these aligned with their perspectives. Feedback from those consulted indicated no necessity for adjustments to the data analysis. In addition to the interviews, a short online survey was conducted and for which data will be reported in full elsewhere.

#### 3.5.2 Results

**(1) Co‐Developed Interventions.** Several strengths and weaknesses of the co‐developed interventions were identified by participants.

The resource directory for patients and their relatives was perceived as a complementary and valuable tool for both clinic staff and patients. Knowing about useful patient resources helps clinic staff feel more equipped to address various issues and guide patients more effectively, as emphasized by a member of the clinic management team:

“Having that is like a [safety] net. It makes us feel better about our actions.”

However, the fact that the number of resources varies across clinics and that the directory requires frequent updates to ensure the information remains up to date were underscored by clinic staff.

Based on interview findings, the medical appointment preparation form was perceived as highly beneficial, notably by enhancing consultation readiness for both patients and staff. This, in turn, reduced mental burden and made appointments more efficient and tailored to patients' needs, as one nurse explained:

“Given that we sometimes have limited time with patients in clinical settings, this allows us to maximize time so that patients get more out of their visit.”

The form also facilitated discussions on a wide range of topics and helped clinic staff identify emotions or concerns in patients that might otherwise go unnoticed. However, its implementation requires adaptation and workflow adjustments for clinic staff. Furthermore, the information shared may raise confidentiality concerns or cause confusion among staff when they are unsure how to handle it. This was notably mentioned by another nurse:

“Sometimes I feel like… like I'm violating someone's privacy by reading the form. It's like I don't have the right to read it. […] I don't know if the patient wants me to read it. […] It made me a little uncomfortable.”

Finally, some staff members expressed concerns about the negative consequences for patients if certain doctors would not consult completed forms, namely that patients might feel that they have wasted their time and that their concerns and needs are not important to their care team.^3^

Regarding the posters promoting discussions on mental health, clinic staff and patients reported that these reflect the staff's openness to discussing mental health and encourage patients to engage in related conversations, as mentioned by one nurse:

“There are definitely patients who looked at the poster and really addressed the subject as a consequence.”

However, some clinic staff felt that this initiative could be perceived as intrusive by patients and create implicit pressure, causing them to feel compelled to discuss their mental health.

Participants also reflected on the strengths and weaknesses of additional interventions implemented by the clinic's management team, specifically those that were in place at the time of the interviews. Regarding the mental health training sessions, they were perceived as valuable and informative by clinic staff, clarifying their responsibilities in supporting patients' mental health. The sessions also helped them feel more confident in their role and reduced feelings of guilt, as emphasized by one member of the clinic management team:

“It's also about learning that yes, you can receive this information, but that doesn't necessarily mean it's up to you to act […] as long as afterwards you orient them properly.”

These sessions also encouraged the sharing of challenging experiences among colleagues and increased awareness of patients' psychological distress. Finally, clinic staff found the guidelines developed for mental health referrals highly useful, facilitating several mental health referrals within the healthcare system. This was notably emphasized by one doctor:

“It has also clarified the path and the possible resources for everyone in fact. Before, it was very vague. It has allowed us to really have practical resources.”

However, significant delays in accessing resources and services remain a major challenge.

**(2) Participatory Research Process.** Participants expressed highly positive opinions regarding the participatory research process. Both patients and staff appreciated the sharing of perspectives facilitated by the project and found that this provided a richer and more nuanced understanding of the complex issue at hand. This was notably echoed by this patient living with a rare immunological disease:

“A real exchange [between patients and HCPs] in the healthcare environment, there aren't that many. This is a project that I think can help with that.”

Participants also appreciated that the project offered dedicated time and space to explore and address the issue. Some also reported that the process encouraged constructive introspection on the quality of care with a focus on continuous improvement. Regarding impact, participants reported that the participatory research process facilitated the development of interventions more closely aligned with the needs of patients and clinic staff, largely due to ongoing stakeholder feedback, which enhanced the successful implementation and acceptance of these interventions, as stated by one member of the clinic management team:

“It engages people more afterwards [after the project] since the solutions come from themselves. It's a lot less frustrating than having ideas imposed on you by a management team that isn't in touch with day‐to‐day life.”

Regarding stakeholder involvement in the process, the feeling of being heard, involved, and contributing to positive change was seen as empowering by some. Furthermore, some felt that the research team's neutrality allowed for a freer expression of needs and encountered difficulties related to the issue. Finally, this living lab participatory approach was seen as helpful and innovative in addressing issues encountered in clinical settings.

**(3) Project's Impacts**. Following a living ethics stance [10], the project's impact on co‐learning, co‐imagination, dialogue, flourishing, and empowerment was explored with participants.

#### 3.5.3 Co‐Learning

The project deepened patients' understanding of the challenges in providing mental health support and concrete changes, as emphasized by one patient living with a rare immunological disease:

“I also realized how difficult it is to tackle certain questions. When you're a patient, you find that everything is perhaps simpler. But in reality, it's not as… There are lots of limits. There are lots of… It's complicated to address this kind of issue.”

For clinical staff, the project expanded their perspectives on the potential of such initiatives and the complexity of driving concrete changes. They also learned about themselves, their colleagues, the issue of patients' psychological distress, and potential avenues for addressing it.

#### 3.5.4 Co‐Imagination

The project encouraged reflection and raised awareness among patients about this issue beyond their personal experiences, while fostering creativity and imagination in developing tailored and appropriate solutions, as emphasized by this patient living with a rare immunological disease:

“We couldn't just say, ‘Okay, well the person is in psychological distress, let's refer them to a psychologist.’ You know? We really had to go beyond certain conventional practices.”

For clinical staff, it prompted introspection on improving care and practices, while also highlighting additional challenges. It also shifted their perception of the phenomenon, enhancing their recognition of more subtle signs of psychological distress.

#### 3.5.5 Dialogue

The project encouraged patients to discuss mental health with their circle and care team. Among clinical staff, it opened and facilitated discussions with colleagues, as emphasized by this doctor:

“It has allowed to free up the conversation about it, because it's not something we talked about much. It's something we experience every day, but without it really being identified or discussed.”

The project also improved dialogue with patients, not only about mental health but also on broader topics.

#### 3.5.6 Empowerment and Flourishing

The project's impact on empowerment and flourishing overlap in several ways as it appears to have fostered, or even increased, participants' agency in various respects, which ultimately enhanced the well‐being and sense of flourishing of some. For instance, patients valued the opportunity to express themselves, feel heard, and contribute meaningfully despite illness‐related constraints, which reduced feelings of helplessness and fostered a sense of utility. Among clinical staff, the project acted as a catalyst for prioritizing and implementing concrete interventions, which would have been challenging without the project's data, framework, deadlines, and resources. This was notably mentioned by a member of the clinic management team:

“It also allowed… to just create momentum. […] It enabled us to prioritize issues that we wouldn't have, that we wouldn't have tackled. First, because we hadn't done the analysis [of the issue] and because we didn't have everything we needed to do it. But now, we had everything we needed. And it acted as a lever to set up other complementary interventions to the project. So, it really had a leverage effect on our ability to put things in place.”

As a result, the project equipped clinic staff with better tools to address patients' psychological distress, enhancing their sense of control and confidence, and reducing feelings of guilt. This was notably echoed by one nurse:

“It's kind of soothing, in the sense that now we have something that makes it easier. It becomes easier to discuss subjects with patients with less guilt. You feel you're doing more for the patient.”

(**4) Challenges and Limitations of the Project.** Participants identified several challenges related to the project. These included actively engaging in the project, the complexity of the issue at hand, and transitioning from testimonials to actionable, realistic, cost‐effective, and high‐impact interventions that could be implemented quickly. Balancing the diverse expectations of stakeholders and managing the short timeline imposed by research funding were also significant obstacles. Finally, concerns were raised about sustaining interventions and progress beyond the project's conclusion, as emphasized by one clinic staff member:

“But you know, we don't have the time to keep it going as a full‐time research project like you do. So, I think that's the challenge, to see how we can continue it without it being too demanding.”

As for limitations, an important one was the project's inability to fully address the issue on its own or shift deeply rooted mentalities regarding mental health among some staff members. Additionally, variability in stakeholder engagement and significant challenges in accessing mental health resources within the healthcare system were noted as barriers to achieving meaningful change. In this regard, a member of the clinic management team commented:

“It's all good to identify and then have resources, but we have a problem in the healthcare system with access to mental health services. So, we're somewhat limited by what we can do, because it's above our heads.”

Some participants also pointed out that potential biases might have arisen from the specific sample of participants, which could have influenced both the understanding of the issue, the selection of interventions, and the evaluation of the project. Another limitation was the lack of direct interactions between patients and staff, as some staff preferred to maintain distance to preserve future relationships with patients and to express their views more freely. Finally, participants identified limitations related specifically to the research context in which this initiative was carried out. These included the restricted timeline and financial resources dictated by the research funding, which made it impossible to hire a mental health specialist at the clinic, as well as the distance between stakeholders and the executive team due to fragmented and occasional communication.

## 4 Discussion

Psychological distress associated with living with a complex or rare chronic disease is a common but nonetheless difficult experience for patients [32]. Its management by specialized HCPs is often suboptimal because of their focus on the complex biomedical features of illness and because of barriers in access to primary and mental healthcare [4, 6, 7]. Consequently, the nonphysical dimensions of the patient's condition can easily be neglected despite the consequences and implications of unaddressed psychological distress. Living ethics is a newly proposed ethics stance which brings attention to everyday moral problems and encourages action‐oriented participatory research and innovation processes to address them [9, 10]. In line with this orientation, we undertook a living ethics project which borrowed the methodological form of a living lab to address the issue of unaddressed psychological distress experienced by people living with complex or rare chronic diseases. This project engaged patients and staff from a specialized interdisciplinary care clinic over 2 years, across five project phases. The problem identification phase (phase 1) led to the identification of this issue perceived as concerning and important by both patients and clinic staff. The subsequent problematization phase (phase 2) shed light on central dimensions of the issue (e.g., how patients and clinic staff conceptualize patients' psychological distress, its causes, consequences, mitigating factors, management within the healthcare framework, and potential avenues for improvement). Providing a deeper understanding of the phenomenon, these findings laid the groundwork for co‐imagining potential interventions to improve the management of patients' psychological distress at the clinic. During the co‐ideation phase (phase 3), 17 potential interventions were suggested and explored with stakeholders, leading to the prioritization of three interventions and the subsequent enactment phase (phase 4): a resource directory, a medical appointment preparation form, and posters promoting discussions on mental health. The project's momentum also led clinic's management team to take on four additional interventions in collaboration with the research team: offering suicide prevention training to clinic staff, developing official mental health referral guidelines, creating a web page with mental health resources for patients, and producing a card listing general mental health resources for patients. Finally, the interventions, research process, outcomes of the project, as well as its challenges and limitations were ultimately assessed by stakeholders during the evaluation phase (phase 5).

As one of the first attempts to both find inspiration in living ethics as a distinctive stance [9, 10] and undertake a living lab in ethics, this project was a perilous enterprise plagued by several methodological and practical unknowns. Surprisingly, it was able to deliver on both fronts though not without challenges and limitations. In the following, we comment on the strengths and challenges of this living lab project in ethics, as well as on the implications of our findings for a living ethics stance.

### 4.1 Strengths and Challenges of This Living Lab Project in Ethics

Overall, this project proved a success. Our results show that deploying a living lab methodology in ethics provides an in‐depth, polyphonic understanding of the moral issues experienced by patients and HCPs, which in turn enables the consideration of a wide range of tailored interventions directly liked to and responsive to the various dimensions of the issue at hand (i.e., its causes, consequences, and mitigating factors). For instance, a closer examination of the co‐developed interventions reveals that they were oriented toward three key objectives: (1) better equipping the clinic staff with mental health resources (as was the case for the resource directory, suicide prevention training, and mental health referral guidelines), (2) enhancing information sharing and communication between patients and staff (as was the case for the preparation form and the posters promoting discussion on mental health), and (3) integrating mental health into the medical follow‐up (as was the case particularly for the medical appointment preparation form, though all interventions addressed this objective in some way). These objectives directly relate to the findings from the problem identification phase, which revealed gaps in staff training and skills related to mental health, the positive impact of information on alleviating patients' psychological distress, and the suboptimal integration of mental health at the clinic. These results thus support the relevance of a participatory, open, iterative, and action‐oriented research methodology in ethics—such as a living lab—where time is devoted to expressing and deeply understanding moral issues, the perspectives of multiple stakeholders are considered and oriented toward finding solutions, and interventions are suggested by the stakeholders themselves in response to perceived needs. In the reported project, this resulted in the co‐development of interventions that were concrete, low‐cost, and easy‐to‐implement, despite the complexity of the problem at hand. Moreover, these interventions were successfully integrated into practice and were favorably evaluated by stakeholders. These results thus powerfully show that complex problems do not always call for complex solutions.

If we now turn to stakeholders' appreciation of the project, our results further suggest that a living lab project in ethics can generate strong stakeholder involvement, as evidenced by the high participation rate throughout the project and the very favourable evaluation of the participatory process by participants. In this respect, participants notably appreciated that the project created a dedicated space and time to address this issue, enabled the sharing of perspectives, fostered constructive introspection on the quality of care, and led to interventions that met their needs and were thus more easily adopted. Moreover, the fact that additional interventions were deployed by the clinic's management team suggests that such a project can create momentum and foster collective dynamics, leading to the ethical issue being addressed from various angles and through diverse efforts. These findings thus challenge the traditional view of research as burdensome for participants, particularly in the context of participatory research approaches in ethics. Yet participatory research remains a rather marginal approach in bioethics, in contrast to its long‐standing history in fields such as social work and education [33]. Hence, a participatory methodology like living labs holds promise for bringing ethics closer to life and action.

Nevertheless, this project was not without challenges and limitations, as highlighted by stakeholders and as we experienced as a research team. Key obstacles included the difficulty for stakeholders to remain engaged throughout this 2‐year project, balancing expectations when co‐imagining potential interventions, sustaining progress after the project's end, overcoming limited access to mental health resources, and the constraints of research funding, timelines, and fragmented communication. The first phase of the study was also modest and organized as a form of consultation to build the research protocol (e.g., a phase of consultation permitted without the need for ethics approval to prepare the study but without the permission to undertake data gathering). The number of participants was small given our tight timeline and modest resources. It is possible that consultation with a broader number of participants would have yielded additional ideas for the focus of the project. Still, the results clearly support the value of participatory methods such as living labs in ethics research and the headway made regarding the selected topic was appreciated positively overall.

### 4.2 Implications for a Living Ethics Stance

This project was not only a living lab project, but also one inspired by a living ethics stance [9, 10], hence the designation *living ethics project*. The results obtained regarding the project's ethical outcomes in line with the methodological guideposts of living ethics suggest that the latter did, in several respects, enact a living ethics stance. First, it was experientially grounded as it addressed an issue experienced and identified by stakeholders themselves. The project also positively impacted co‐learning, co‐imagination, dialogue, empowerment and flourishing among stakeholders. Regarding co‐learning, it provided valuable learning opportunities for both patients and clinical staff, particularly about the issue at hand and the complexity of addressing it. This, in turn, fostered creativity, co‐imagination, and introspection in thinking about appropriate solutions. By bringing the issue of patients' psychological distress to the fore, the project also encouraged and facilitated dialogue on this topic and more broadly on other aspects of patients' experiences. Finally, with respect to empowerment and flourishing, patients reported feeling heard, valued, and empowered by their contributions to meaningful changes, which enhanced their well‐being. Clinical staff, in turn, gained confidence and felt better equipped to address this issue thanks to the interventions, which also reduced feelings of guilt. Thus, the living ethics stance that guided this project, not only through the chosen methodology but also more broadly through the research team's approach, undoubtedly contributed to its success. This provides early evidence in support of this ethics orientation and its potential transformative benefits in clinical settings. However, another living ethics initiative conducted in parallel in another clinical setting led to entirely different outcomes with mitigated impact and engagement (see [20] for more details). This suggests that the success of living ethics projects is not guaranteed but depends on various factors, which are currently being explored and will become clearer as this orientation is trialled in other settings and by different research teams. Nonetheless, the very positive outcomes of this project seem to indicate that this stance holds promise for ethics research and practice to serve as a catalyst to improve healthcare.

## 5 Conclusion

In this paper, we report on the process and outcomes of a living ethics project, structured as a living lab, on the psychological distress of individuals living with a complex or rare chronic disease. We provided an integrated overview of the project's five phases, while focusing on the later phases, particularly on the interventions co‐developed, the project outcomes, and their evaluation by stakeholders. To our knowledge, this is the first study to approach psychological distress in the context of chronic disease from an ethical perspective, using a participatory and iterative approach such as the living lab methodology. Our project has revealed that this issue is considered important and concerning not only by patients, but also by HCPs who often feel ill‐equipped to address it. The process also led to a polyphonic, in‐depth understanding of the issue, resulting in simple interventions that were rapidly integrated into practice and evaluated favorably by stakeholders. Overall, this study shows that directly engaging stakeholders in ethics research, by addressing the moral issues they deem significant and working with them to tackle those issues rather than conducting research on them, can lead to tangible, unexpected, and positive moral and clinical outcomes, even within a short timeframe and with limited resources. These results thus exemplify the potential of a living ethics stance, that is, a stance that brings ethics closer to everyday life and builds on collaboration, co‐learning, dialogue and action to foster stakeholder empowerment and flourishing. Nevertheless, given that another living ethics project conducted in parallel did not yield results as promising as these, further initiatives across diverse contexts will be essential to better understand the conditions under which this stance can meaningfully contribute to ethics research and practice.

Draft versions of the text were translated from French to English using *DeepL*, and then thoroughly revised and refined by the authors who are fully bilingual to ensure that the wording accurately conveyed their intended meaning.

## Author Contributions

**Bénédicte D'Anjou:** conceptualization, data curation, formal analysis (lead), investigation, methodology, project administration, supervision, visualization, writing – original draft preparation (lead). **Katherine Desjardins:** investigation, resources, validation, writing – review and editing. **Julie Ianniruberto:** investigation, methodology, validation, writing – review and editing. **Danielle Méthot:** investigation, resources, validation, writing – review and editing. **Valérie Poulin:** investigation, resources, validation, writing – review and editing. **Rémi Rabasa‐Lhoret:** conceptualization, funding acquisition, investigation, resources, validation, writing – review and editing. **Eric Racine:** conceptualization, funding acquisition; formal analysis, investigation, methodology, project administration, supervision (lead), writing – original draft preparation (supporting).

## Ethics Statement

A complete research protocol was developed and submitted for ethics review (approval no. 2023‐1205 from the Research Ethics Board of the Montreal Clinical Research Institute (IRCM)). All participants provided content.

## Conflicts of Interest

The authors declare no conflicts of interest.

## Supporting information

Supplementary File Medical Appointment Preparation Form Word.

Supplementary File Mental Health Poster 1 Word.

Supplementary File Medical Appointment Preparation Form Word. Supplementary File Mental Health Poster 1 Word. Supplementary File Mental Health Poster 2 Word. Supplementary File Mental Health Resource Directory Word.

Supplementary File Mental Health Resource Directory Word.

## Data Availability

The authors have nothing to report.

## Endnotes

1 During the later phases of the project, the clinic recruited a collaborative project coordinator (V.P.) who subsequently joined the project team.

2 As the web page and the resources card were not yet available at the time of the evaluation phase, they could not be evaluated by participants.

3 The survey results – to be reported in full elsewhere – showed an overall positive appreciation of the medical appointment preparation form by clinic patients. Most respondents (70%) out of 267 who completed the survey reported that the form helped them prepare for their medical appointment. Additionally, 66% indicated that the form facilitated discussions about their physical health, and 55% felt it helped discussions on psychological health and well‐being. In terms of overall quality of the appointment, 62% of respondents agreed that the form contributed to improving their experience. Looking ahead, 80% stated they were likely to complete the form before their next appointment.
