# Supplementary material for: A Living Ethics Project to Address Psychological Distress in Chronic Illness: Process and Outcomes
Source: Health Expect. 2025 Dec 17;28(6):e70457. doi: 10.1111/hex.70457 (PMC12710513; doi:10.1111/hex.70457)
Supplement: Supplementary file 1 — Supplementary File Medical Appointment Preparation Form Word. [file HEX-28-e70457-s001.docx]

Ce formulaire a été développé par l’Unité de recherche en éthique pragmatique de la santé dans le cadre d’un projet de laboratoire vivant É-LABO, réalisé à l’Institut de recherches cliniques de Montréal (IRCM) avec le soutien financier du ministère de l’Économie, de l’Innovation et de l’Énergie et des Fonds de recherche du Québec – Santé. Si vous souhaitez utiliser ou reproduire, en tout ou en partie, ce document, veuillez communiquer avec le Dr Eric Racine à l’adresse suivante : [eric.racine@ircm.qc.ca.](mailto:eric.racine@ircm.qc.ca)


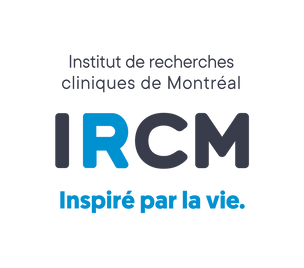

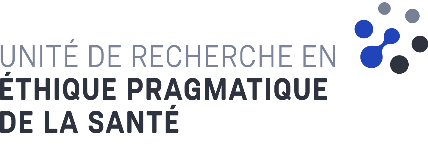

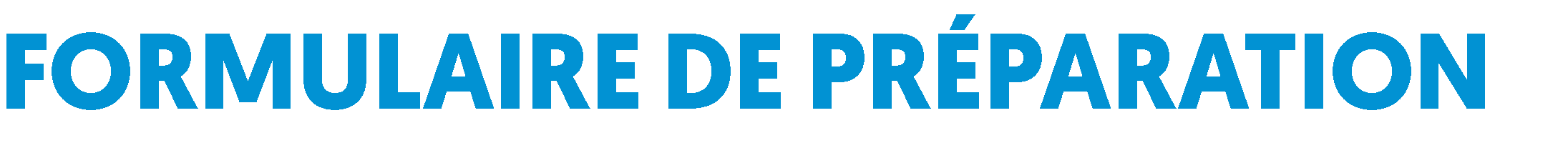

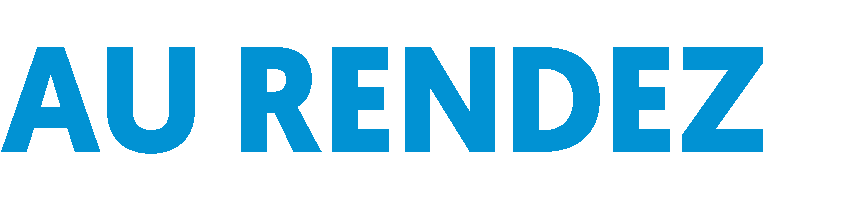

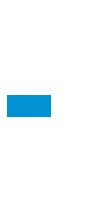

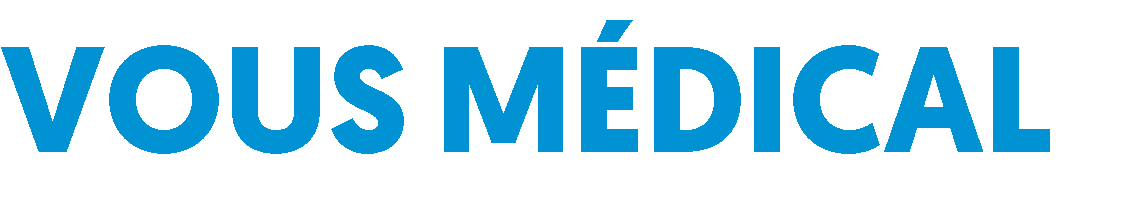


**Ce formulaire, facultatif et confidentiel, vise à améliorer la qualité de votre suivi médical. Vos réponses nous aideront à préparer votre rendez-vous et à adapter votre prise en charge en fonction de ce qui compte le plus pour vous.**

1. Avez-vous vécu ou constaté des changements au niveau de votre santé depuis votre dernier rendez-vous médical (p. ex. : nouveaux symptômes, traitement, suivis médicaux) ?
2. Quels sont les sujets ou les questions que vous aimeriez aborder lors de votre prochain rendez- vous médical (p. ex. : gestion de la maladie, qualité de vie, traitements, inquiétudes, etc.) ?
3. Qu’est-ce qui vous préoccupe le plus à propos de votre santé physique et psychologique ?
4. À la clinique de l’IRCM, nous prenons à coeur la santé mentale de nos patient∙e∙s. Nous sommes là pour vous soutenir au meilleur de nos capacités et pour vous orienter vers les ressources appropriées. Sur une échelle de 1 à 10, comment se porte votre santé psychologique ces jours-ci ?

| 1 | 2 | 3 | 4 | 5 | 6 | 7 | 8 | 9 | 10 |
| --- | --- | --- | --- | --- | --- | --- | --- | --- | --- |

Nous vous remercions d’avoir rempli ce formulaire de préparation au rendez-vous médical. Sachez que vous pourrez toujours discuter d’autres sujets que ceux mentionnés dans ce formulaire avec votre équipe médicale si vos besoins changent d’ici votre rendez-vous. Avez-vous d’autres commentaires ou questions à partager à votre équipe médicale d’ici votre rendez-vous ?

Ce formulaire a été développé par l’Unité de recherche en éthique pragmatique dans le cadre d’un projet de laboratoire vivant É-LABO, réalisé à l’Institut de recherches cliniques de Montréal (IRCM) avec le soutien financier du ministère de l’Économie, de l’Innovation et de l’Énergie et des Fonds de recherche du Québec – Santé. Si vous souhaitez utiliser ou reproduire, en tout ou en partie, ce document, veuillez communiquer avec le Dr Eric Racine à l’adresse suivante : [eric.racine@ircm.qc.ca.](mailto:eric.racine@ircm.qc.ca)


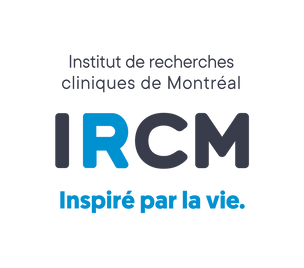

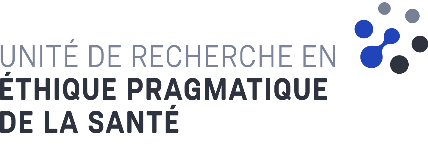

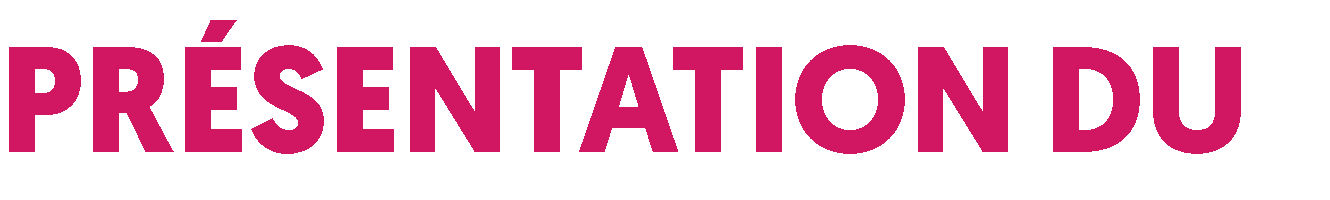

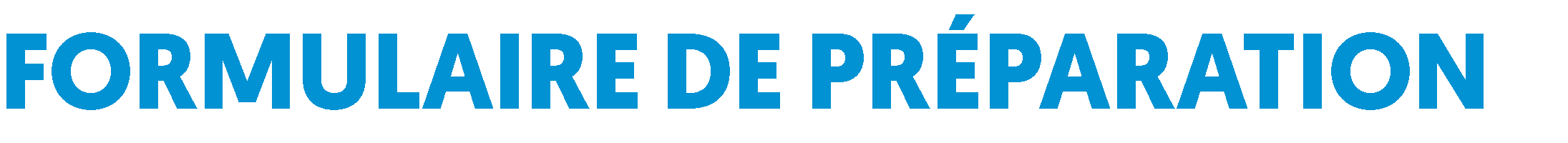

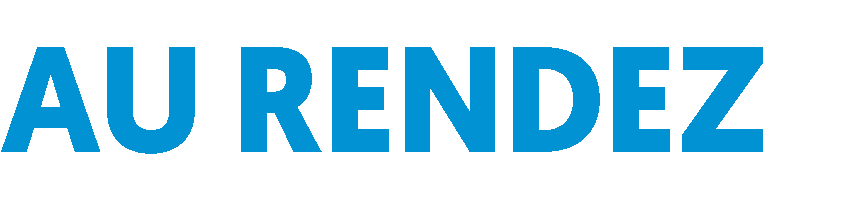

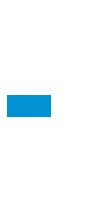

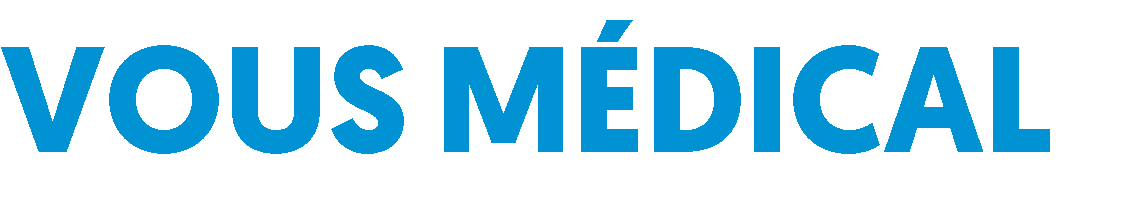


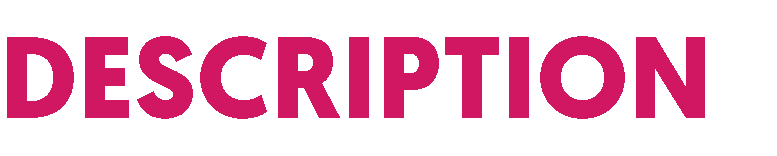


Ce formulaire facultatif vise à promouvoir une prise en charge plus globale et personnalisée de votre santé. Il vous permet d’informer votre équipe médicale des événements de santé importants survenus depuis votre dernier rendez-vous. Cela peut inclure de nouveaux symptômes, diagnostics, traitements, hospitalisations, suivis avec des professionnel∙le∙s de la santé ou tout autre élément que vous jugez pertinent. Il vous invite de plus à indiquer les sujets et/ou questions que vous aimeriez aborder ainsi que vos préoccupations afin que votre suivi médical à la Clinique de l’IRCM soit orienté en fonction de ce qui compte le plus pour vous. En outre, il vous permet d’indiquer comment se porte votre santé psychologique pour donner une meilleure idée à votre équipe médicale de votre état de santé globale et de favoriser la prise en charge de votre bien-être psychologique.


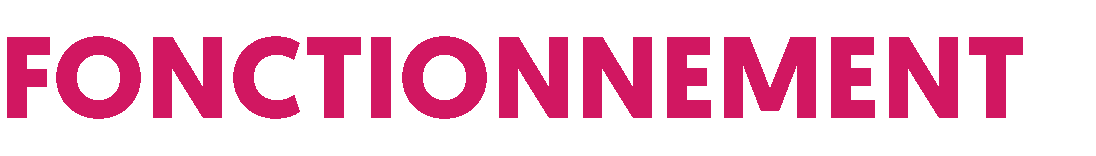


Ce formulaire est facultatif. Il vous est transmis par courriel 5 jours ouvrables avant votre rendez-vous médical, dans le courriel de confirmation du rendez-vous. Nous vous invitons à le remplir dès que possible afin que votre équipe médicale ait le temps nécessaire pour consulter vos réponses et préparer votre rendez-vous en conséquence. Si vous oubliez de remplir ce formulaire ou n’êtes pas en mesure de le faire électroniquement, vous pourrez remplir une version papier de ce formulaire sur place le jour de votre rendez-vous. Ce formulaire vise à orienter votre rendez-vous en fonction de vos besoins. Comme ceux-ci peuvent changer d’ici votre rendez-vous, vous pourrez toujours discuter d’autres sujets que ceux mentionnés dans ce formulaire avec votre équipe médicale.


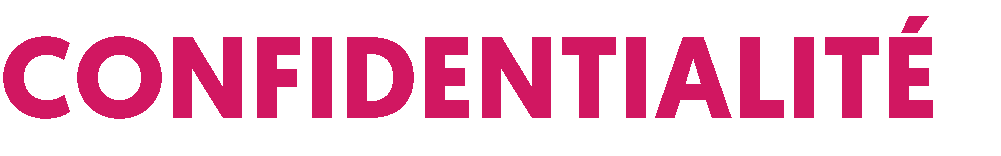


Les réponses à ce formulaire seront enregistrées dans votre dossier médical et accessibles uniquement par votre équipe médicale, conformément aux réglementations sur la confidentialité des données médicales.


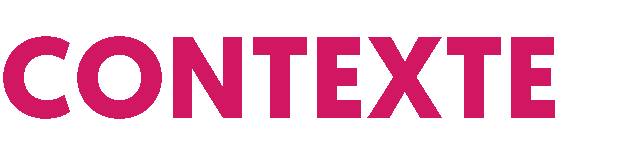

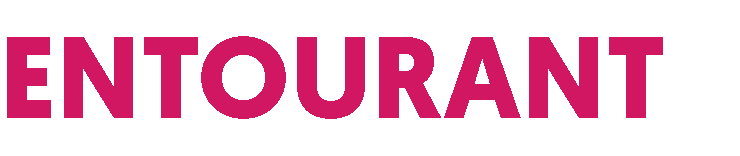

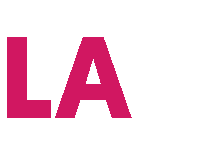

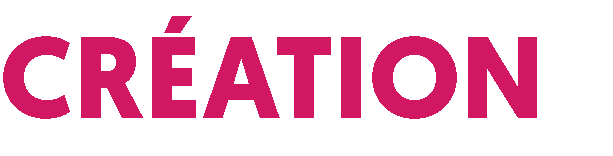

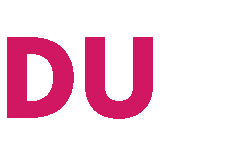

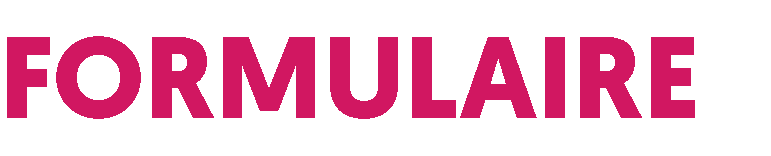


Ce formulaire a été développé par l’Unité de recherche en éthique pragmatique de la santé de l’IRCM dans le cadre d’un projet de laboratoire d’éthique vivante (É-LABO), mené entre 2022 et 2024. Ce projet, inspiré par la recherche-action participative, portait sur la détresse psychologique des patient∙e∙s de la Clinique de l’IRCM. Dans le cadre de cette initiative, les points de vue des membres du personnel et des patient∙e∙s ont été mobilisés afin de mieux comprendre ce phénomène et de mettre en place des interventions visant à améliorer la prise en charge de la santé mentale au sein de la Clinique de l’IRCM. Ce formulaire de préparation au rendez-vous médical est l’une des interventions développées et mises en œuvre dans ce projet. Pour plus d’informations, nous vous invitons à contacter le Dr Eric Racine, chercheur responsable du projet (eric.racine@ircm.qc.ca).
