# Supplementary figures and images for: A Living Ethics Project to Address Psychological Distress in Chronic Illness: Process and Outcomes
Source: Health Expect. 2025 Dec 17;28(6):e70457. doi: 10.1111/hex.70457 (PMC12710513; doi:10.1111/hex.70457)

La santé mentale compte autant que la santé physique.


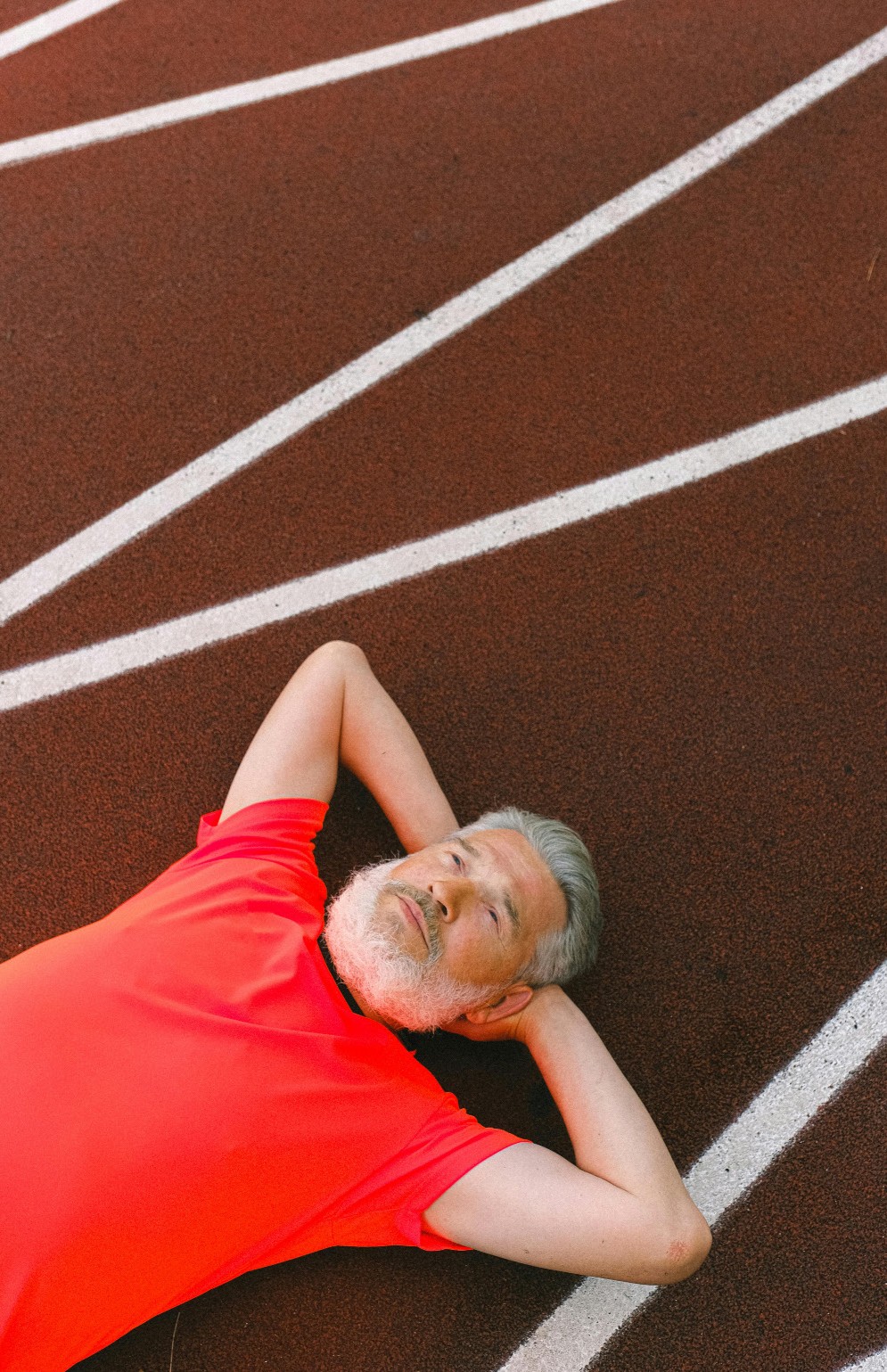

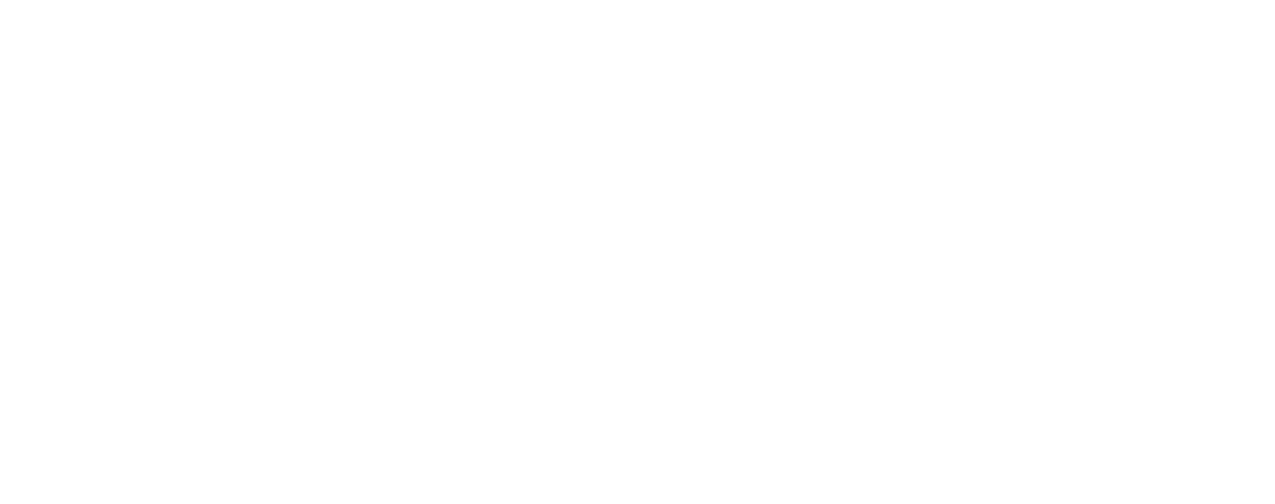

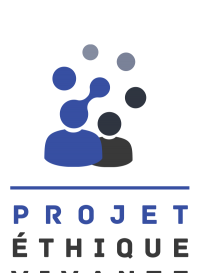


Parlons-en pour rester en forme.

Supplement: Supplementary file 2 — Supplementary File Mental Health Poster 1 Word. [file HEX-28-e70457-s004.docx]

Votre


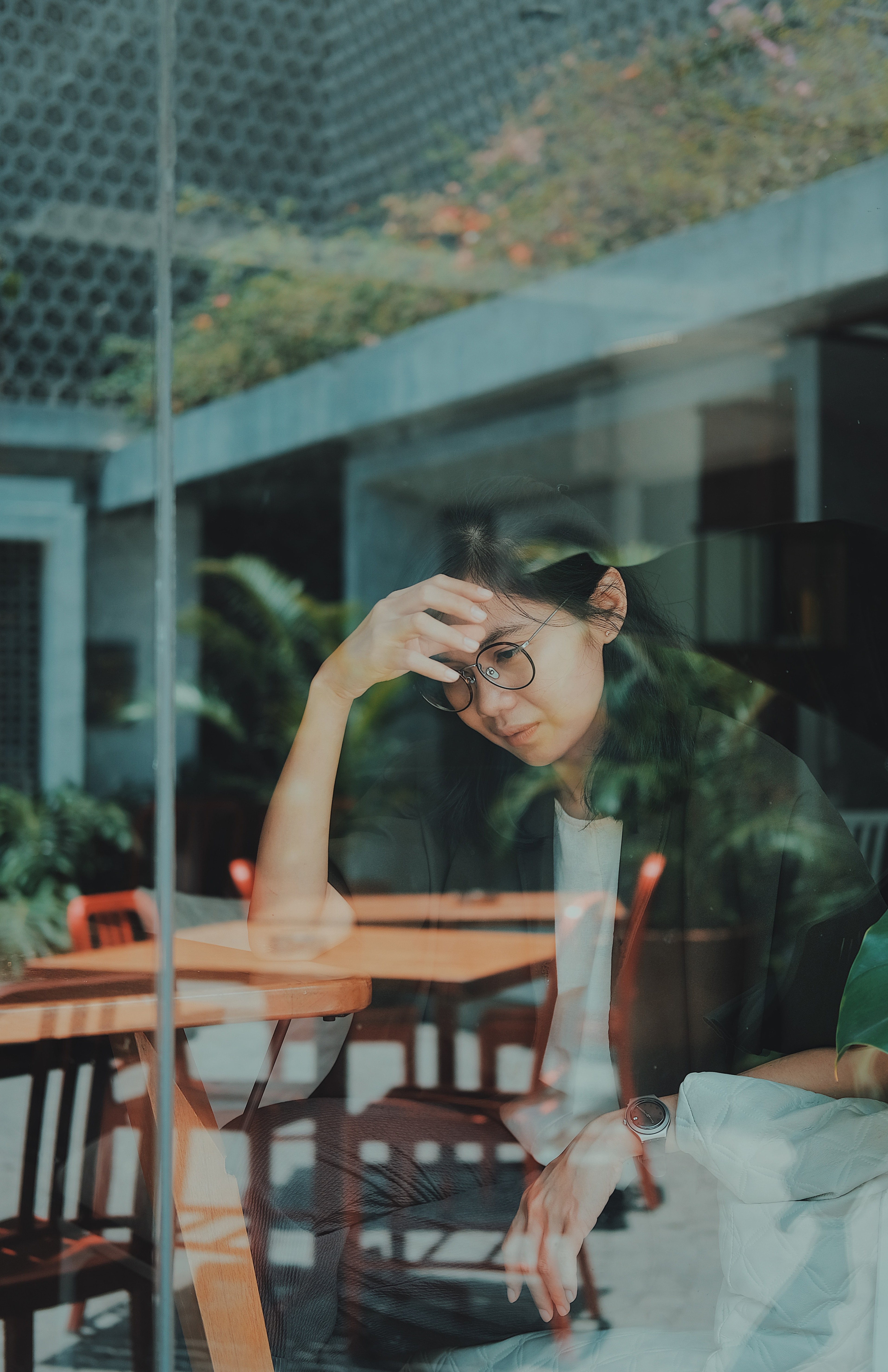

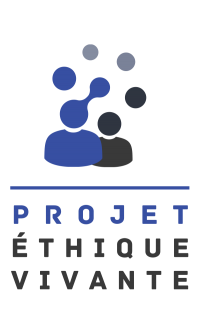

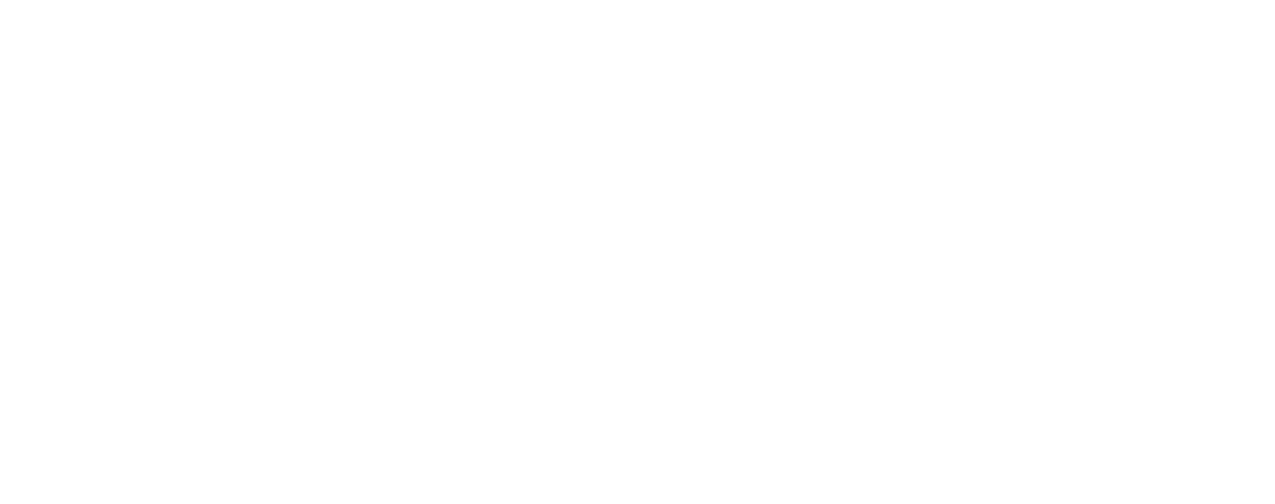


santé mentale mérite d’être abordée.

Parlons-en.

Supplement: Supplementary file 3 — Supplementary File Medical Appointment Preparation Form Word. Supplementary File Mental Health Poster 1 Word. Supplementary File Mental Health Poster 2 Word. Supplementary File Mental Health Resource Directory Word. [file HEX-28-e70457-s002.docx]
