# Supplementary material for: A Living Ethics Project to Address Psychological Distress in Chronic Illness: Process and Outcomes
Source: Health Expect. 2025 Dec 17;28(6):e70457. doi: 10.1111/hex.70457 (PMC12710513; doi:10.1111/hex.70457)
Supplement: Supplementary file 4 — Supplementary File Mental Health Resource Directory Word. [file HEX-28-e70457-s003.docx]

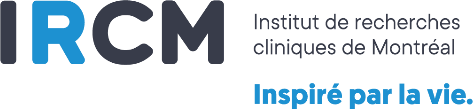

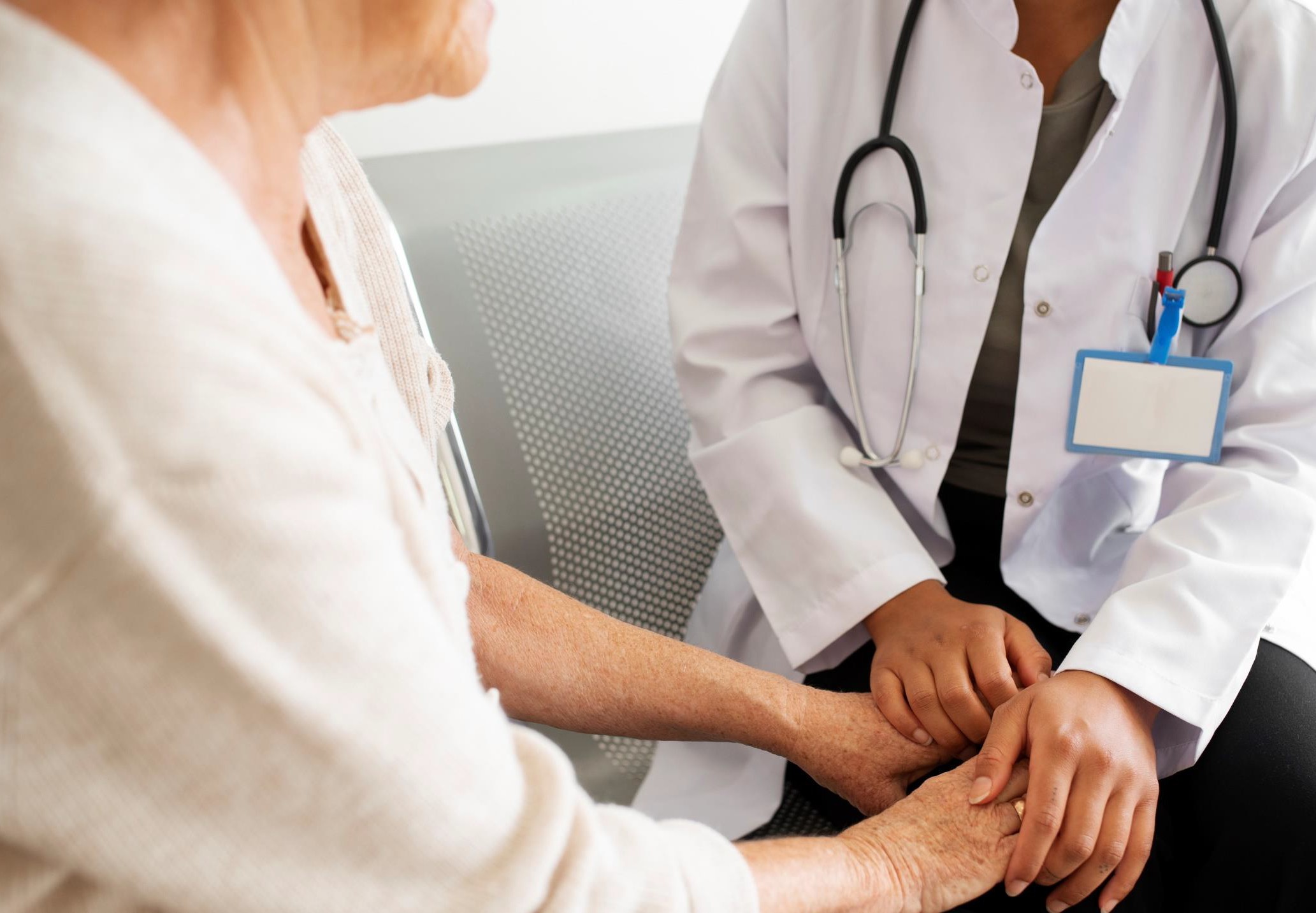

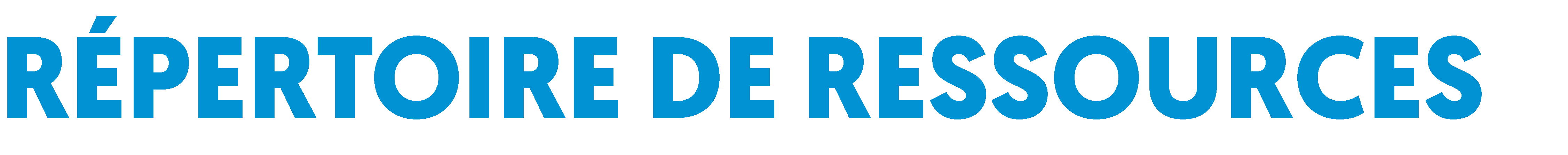

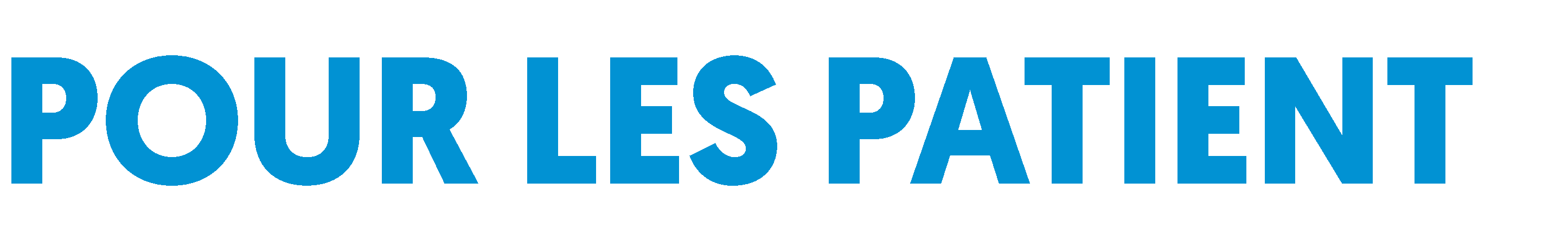

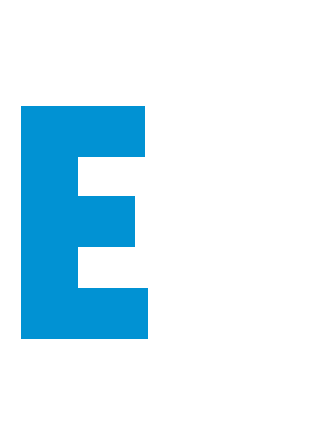

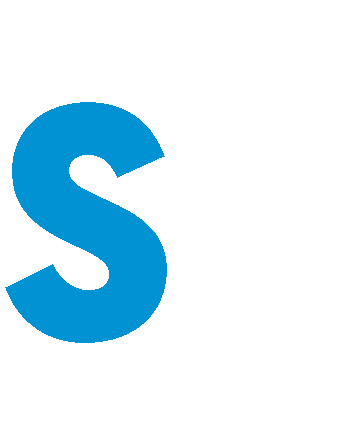

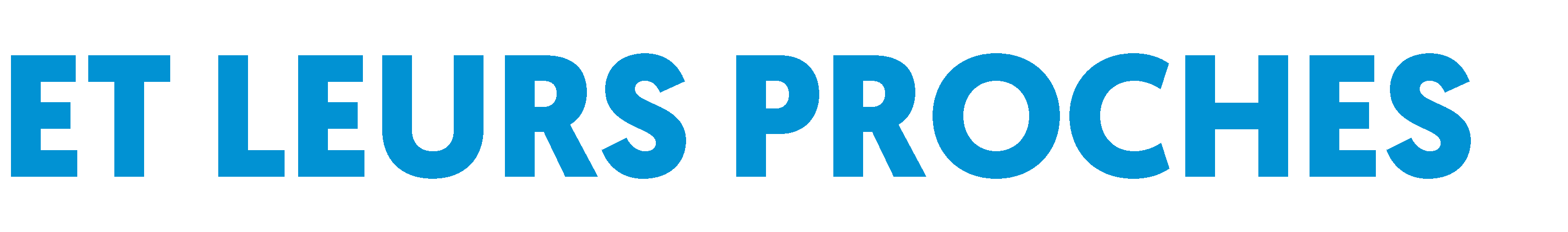

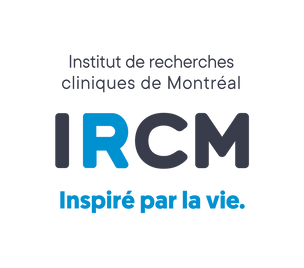

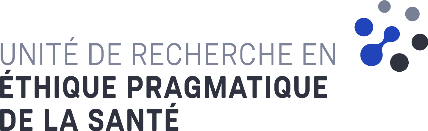


- ∙

Dernière mise à jour : Novembre 2023


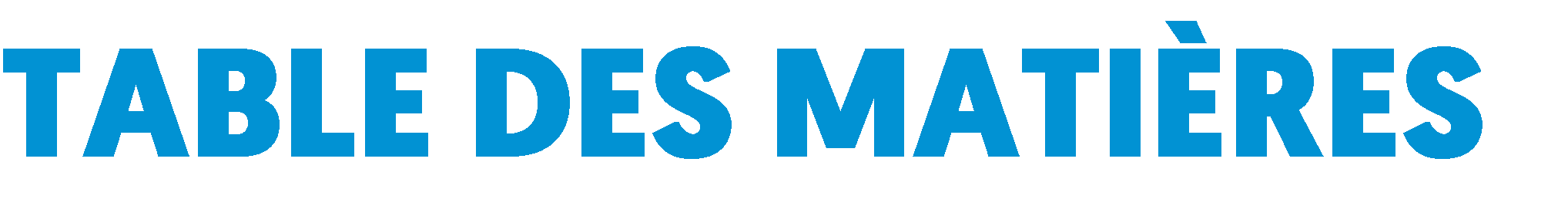


[**À PROPOS DE CE RÉPERTOIRE 2**](#_bookmark0)

[**RESSOURCES POUR LA SANTÉ PSYCHOLOGIQUE 3**](#_bookmark1)

[RESSOURCES GÉNÉRALES 3](#_bookmark2)

[ADAPTATION À LA MALADIE 4](#_bookmark3)

[CONDUITE ALIMENTAIRE 5](#_bookmark4)

[PRÉVENTION DU SUICIDE 5](#_bookmark5)

[**RESSOURCES EN DIABÈTE 6**](#_bookmark6)

[**RESSOURCES EN HYPERTENSION 7**](#_bookmark7)

[**RESSOURCES EN IMMUNOLOGIE 8**](#_bookmark8)

[**RESSOURCES EN LIPIDES 9**](#_bookmark9)

[**RESSOURCES EN MALADIES RÉNALES RARES 10**](#_bookmark10)

[**AUTRES RESSOURCES PERTINENTES 11**](#_bookmark11)

[MALADIES RARES 11](#_bookmark12)

[DROITS DES PATIENT∙E∙S 12](#_bookmark13)

[PROCHE AIDANCE 12](#_bookmark14)


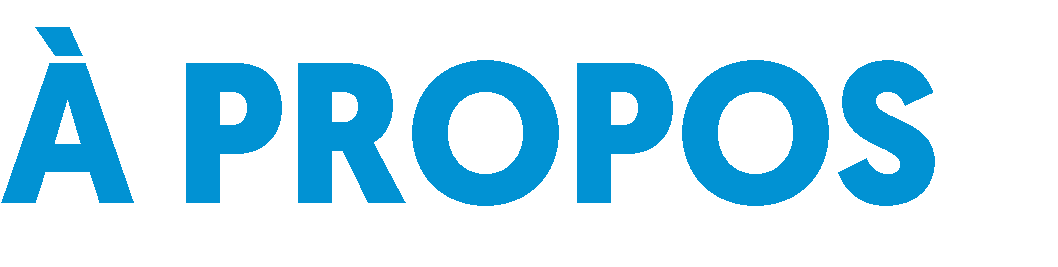

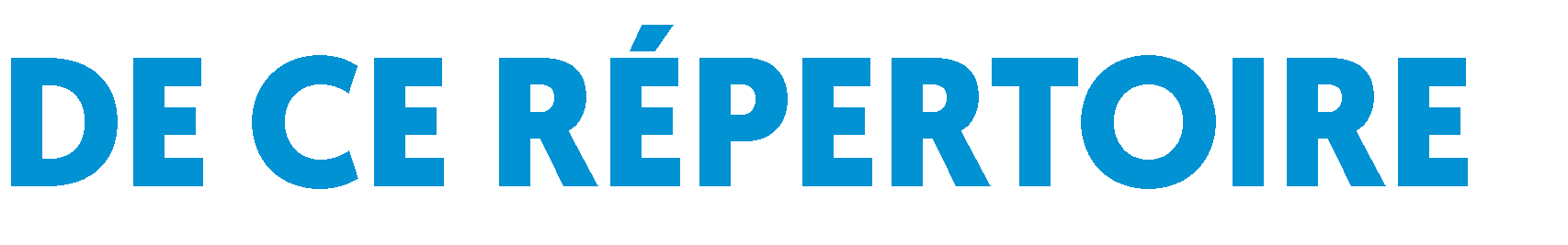


Ce répertoire de ressources pour les patient∙e∙s et leurs proches a été conçu par l’Unité de recherche en éthique pragmatique de la santé. Il s’inscrit dans le cadre d’un projet de laboratoire d’éthique vivante, É-LABO, mené à l’Institut de recherches cliniques de Montréal (IRCM) entre 2022 et 2024 et ayant été financé par le ministère de l’Économie, de l’Innovation et de l’Énergie, ainsi que des Fonds de recherche du Québec – Santé.

Ce projet, inspiré de la recherche-action participative, portait sur la détresse psychologique des patient∙e∙s de la Clinique de l’IRCM. Dans le cadre de cette initiative, les points de vue des membres du personnel et des patient∙e∙s ont été mobilisés afin de mieux comprendre ce phénomène et de mettre en place des interventions visant à améliorer la prise en charge de la santé mentale au sein de la Clinique de l’IRCM. Le répertoire que vous consultez est l’une des interventions développées et mises en œuvre dans ce projet. Il vise à outiller les équipes de la clinique avec des ressources supplémentaires à offrir aux patient∙e∙s et à leurs proches.

Pour toute question ou pour obtenir plus d’informations sur le répertoire, nous vous invitons à communiquer avec le Dr Eric Racine, chercheur responsable du projet, à l’adresse suivante :

[eric.racine@ircm.qc.ca.](mailto:eric.racine@ircm.qc.ca) Si vous souhaitez reproduire ce document, en tout ou en partie, veuillez communiquer avec le Dr Eric Racine à l’adresse mentionnée ci-dessus.


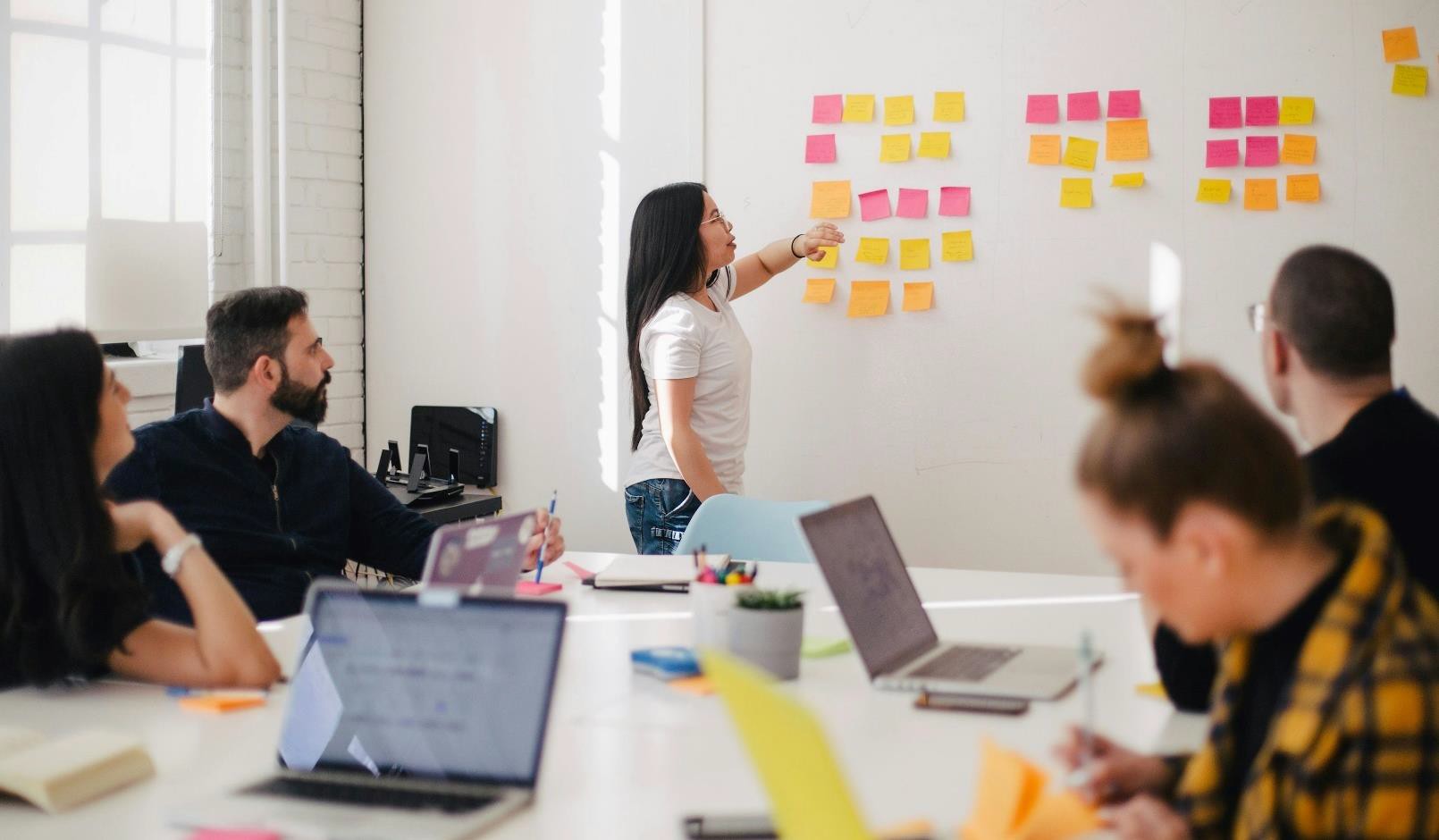


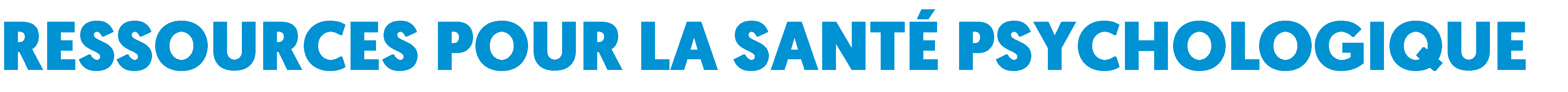


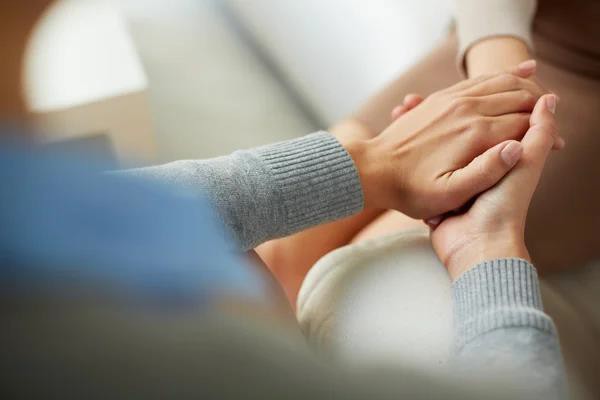


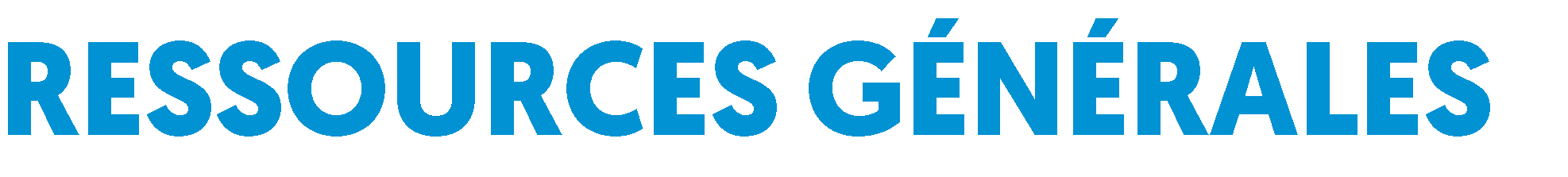


| **Nom et coordonnées** | **Description** | **Population ciblée** | **Région** |
| --- | --- | --- | --- |
| **Info-Social 811**  811 option #2 | - Consultation psychosociale 24 heures/24, 7 jours/7   • Référence vers ressources d’aide au besoin | Grand public | Tout Québec |
| **Ligne 211 Grand Montréal**  211  [www.211qc.ca](http://www.211qc.ca/) | - Service téléphonique ou de clavardage en ligne - Information et références vers les services sociocommunautaires - Répertoire de ressources en ligne | Grand public | Grand Montréal |


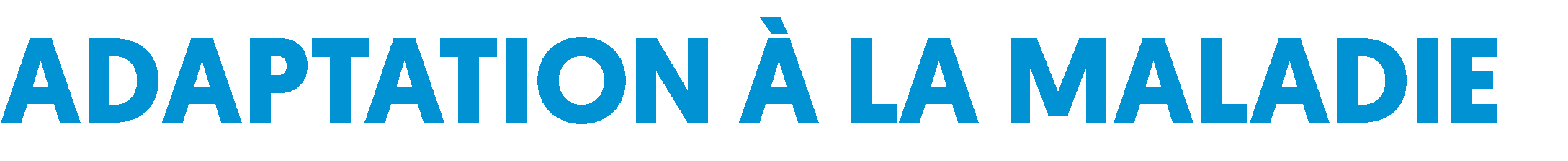


| **Nom et coordonnées** | **Description** | **Population ciblée** | **Région** |
| --- | --- | --- | --- |
| **Anxiety Canada**  [www.anxietycanada.com](http://www.anxietycanada.com/) | - Stratégies et outils pour la gestion de l’anxiété - Thérapie de groupe - Cours en ligne - Application pour l’anxiété à télécharger | Personnes avec symptômes anxieux | Tout Québec |
| **Association canadienne pour la santé mentale**  1-418-529-1979  https://[www.acsmquebec.org](http://www.acsmquebec.org/) | - Promotion de la santé mentale - Prévention et activités de soutien - Centre d'écoute, ateliers psychoéducatifs, conférences et kiosques | Personnes avec des troubles de santé mentale | Tout Québec |
| **Centre de services psychologiques de l’UQAM** 514 987-0253  [www.psychologie.uqam.ca/centre-](http://www.psychologie.uqam.ca/centre-de-services-psychologiques-csp)  [de-services-psychologiques-csp](http://www.psychologie.uqam.ca/centre-de-services-psychologiques-csp) | - Consultation individuelle ou de couple dispensée par des étudiant∙e∙s en psychologie - Les services couvrent les difficultés d'adaptation, l'anxiété, la dépression, les troubles scolaires, les problèmes relationnels, le deuil, etc. | Grand public | Grand Montréal |
| **Centre de soutien en santé mentale**  450 486-1609  [www.cssm-m.com](http://www.cssm-m.com/) | - Intervention à domicile et sur place - Suivi en psychothérapie et psychosocial - Rencontres individuelles ou en groupe - Activités : séances de sport, yoga sur chaise, cours d'anglais, d'espagnol et d'informatique, séances d'art-thérapie, etc. | Personnes atteintes de maladies mentales et leurs proches | Montérégie |
| **Clic Aide**  [www.clicaide.org](http://www.clicaide.org/) | - Consultation en vidéoconférence à faible coût | Personnes, couples et familles vivant une  situation difficile | Tout Québec |
| **Écoute entraide**  1 855-365-4463  [www.ecoute-entraide.org](http://www.ecoute-entraide.org/) | - Groupe de soutien - Ligne d'écoute | Personnes qui recherchent du  soutien | Tout Québec |
| **Espace Mieux-Être Canada (Wellness Together Canada)** Tel1-866-585-0445  Textez MIEUX au 741741  [https://www.wellnesstogether.ca/fr](https://www.wellnesstogether.ca/fr-ca/)  [-ca/](https://www.wellnesstogether.ca/fr-ca/) | - Soutien général en santé mentale - Ligne d'écoute et de support | Adultes désirant du soutien en santé mentale | Tout Québec |
| **G.E.M.E. Groupe d'entraide pour un mieux-être**  [www.groupegeme.com](http://www.groupegeme.com/) | - Groupes de soutien (présentiel et virtuel) - Consultation individuelle (présentiel et virtuel) - Groupes de méditation - Ateliers et conférences - Outils d’autogestion des symptômes | Adultes présentant: stress, phobie, trouble panique, épuisement professionnel, dépression et stress post-traumatique | Tout Québec |
| **La ressource ATP**  (450) 575-4041  [www.laressouceatp.org](http://www.laressouceatp.org/) | - Groupe d'entraide - Formation sur les symptômes anxieux | Personnes ayant un trouble anxieux | Laval |
| **Mouvement santé mentale Québec**  [https://mouvementsmq.ca/qui-](https://mouvementsmq.ca/qui-sommes-nous/membres/)  [sommes-nous/membres/](https://mouvementsmq.ca/qui-sommes-nous/membres/) | - Consultations individuelles - Ateliers de groupe (présentiel et virtuel) | Grand public | Tout Québec |

| **Perspective autonomie en santé mentale**  438 521-7276  [pasm.montreal@gmail.com](mailto:pasm.montreal@gmail.com) | - Écoute et soutien pour personnes vivant des difficultés | Personnes qui présentent toutes problématiques affectant le bien-être  mental | Tout Québec |
| --- | --- | --- | --- |
| **RELIEF**  1-866-738-4873  [www.monrelief.org](http://www.monrelief.org/) | - Groupe de soutien - Accompagnement individuel - Ateliers d’autogestion | Personnes avec symptômes anxieux, dépressifs et  bipolaires | Grand Montréal |
| **Tel-Aide 24h/7**  514 935-1105  <https://telaidemontreal.org/en/> | - Ligne téléphonique d'aide aux personnes ayant besoin d'écoute | Personnes ayant des troubles de santé  mentale | Grand Montréal |
| **Vers l'équilibre**  514 251-1200  [www.verslequilibre.ca](http://www.verslequilibre.ca/) | - Suivi psychosocial individuel et de groupe | Personnes ayant des troubles de santé  mentale | Montréal |


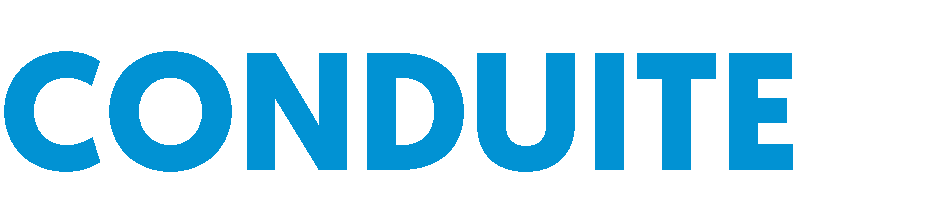

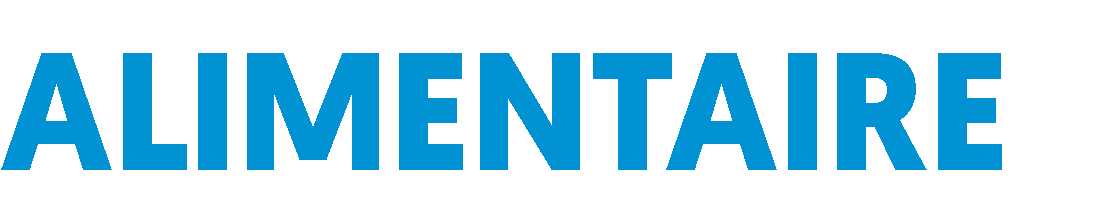


| **Nom et coordonnées** | **Description** | **Population ciblée** | **Région** |
| --- | --- | --- | --- |
| **Anorexie Boulimie Québec**  1 800 630-0907 ou 514 630-0907  [www.anebquebec.com](http://www.anebquebec.com/) | - Ligne d’écoute - Clavarde en ligne - Groupes de soutien - Liste de ressources | Personnes avec préoccupations liées aux comportements alimentaires et leurs  proches | Tout Québec |
| **Équilibre**  1 877 270-3779 ou 514 270-3779  [www.equilibre.ca](http://www.equilibre.ca/) | - Balado, blogue et outils conçus pour favoriser le développement d’une image corporelle positive   ainsi que de créer des environnements inclusifs | Personnes préoccupées par  leur image corporelle | Tout Québec |
| **Maison l'Éclarcie**  1-866-900-1076  [www.maisoneclarcie.qc.ca](http://www.maisoneclarcie.qc.ca/) | - Dépistage sur la conduite alimentaire - Écoute téléphonique et clavardage - Documentation - Rencontres d’accompagnement - Groupes de soutien | Personnes avec préoccupations liées aux comportements alimentaires et leurs proches | Tout Québec |


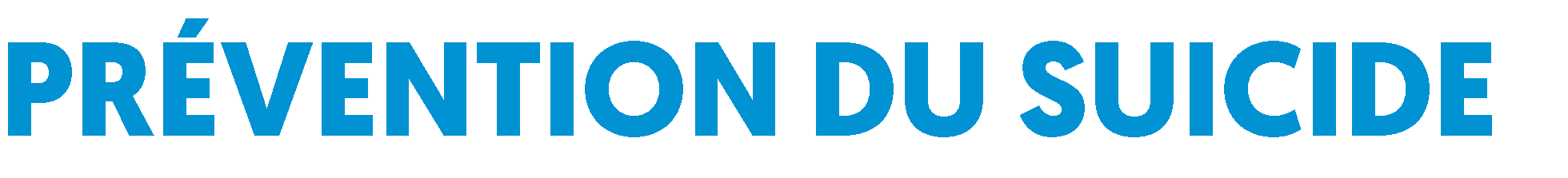


| **Nom et coordonnées** | **Description** | **Population ciblée** | **Région** |
| --- | --- | --- | --- |
| **Association québécoise de prévention du suicide**  1 866 APPELLE ou 988  Texto :535353  [www.suicide.ca](http://www.suicide.ca/) | - Ligne d’écoute - Intervention par clavardage et par texto - Information pour les proches, les personnes endeuillées ou vulnérables au suicide - Liste de ressources | Grand public | Tout Québec |
| **Centre de prévention du suicide de Montréal**  1 866 APPELLE (277-3553)  Texto: 535353  [www.cpsmontreal.ca](http://www.cpsmontreal.ca/) | - Ligne d'écoute - Clavardage en ligne et par texto - Information et outils - Formations et ateliers préventifs | Grand public | Tout Québec |


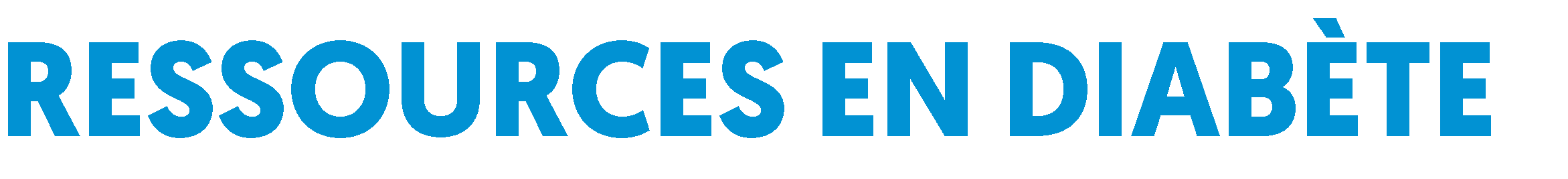


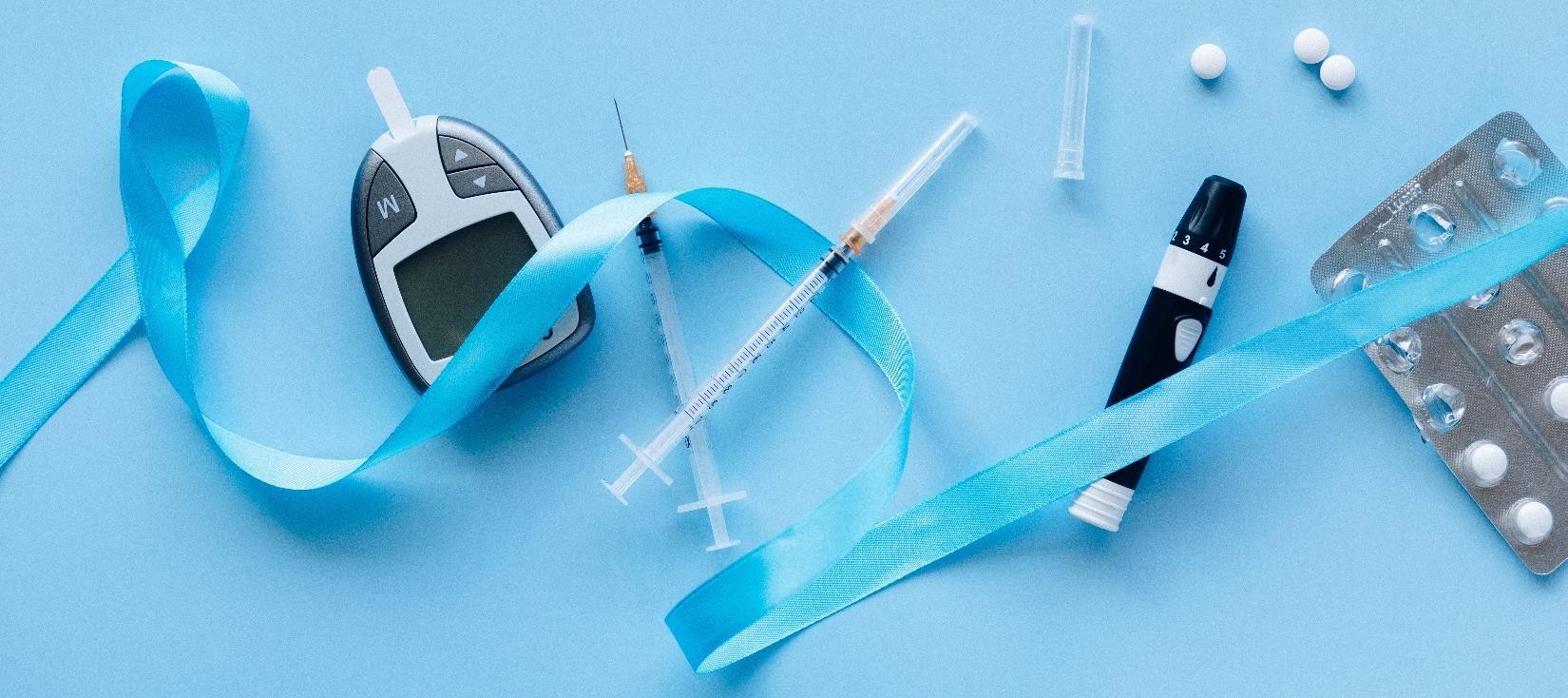


| **Nom et coordonnées** | **Description** | **Population ciblée** | **Région** |
| --- | --- | --- | --- |
| **Amicale des diabétiques du CHUM**  514 890-8000 poste 25358  [www.amicaledesdiabetiquesduchu](http://www.amicaledesdiabetiquesduchum.ca/)  [m.ca](http://www.amicaledesdiabetiquesduchum.ca/) | - Activités: aquaforme, remise en forme, natation, quilles et cours de cuisine - Groupes de soutien - Initiatives liées à la nutrition - Prestation de services de soin des pieds - Dépistage du diabète | Personnes diabétiques | Grand Montréal |
| **Better**  514 987-5696  <https://type1better.com/fr/> | - Webinaire sur le diabète de type 1 - Guide d’information - Guide pour la gestion de l’anxiété - Plateforme de support | Personnes atteintes du diabète de type 1 | Tout Québec |
| **Diabète Québec**  1 800 361-3504 poste 233  [www.diabete.qc.ca](http://www.diabete.qc.ca/) | - Soutien aux personnes souffrant de diabète - Défense des intérêts et des droits - Service d’information - Listes de ressources par région du Québec | Personnes diabétiques, leurs proches et professionnel∙le∙s de  la santé | Tout Québec |
| **Fondation de la recherche sur le diabète juvénile (FRDJ)**  1 877-287-3533  [www.frdj.ca](http://www.frdj.ca/) | - Liste de ressources - Trousse de soins pour enfants et adolescent∙e∙s - Formations pour professionnel∙le∙s de la santé | Personnes diabétiques, professionnel∙le∙s de la santé, grand  public | Tout Québec |
| **Groupe Facebook de soutien au DT1**  [https://www.facebook.com/groups/](https://www.facebook.com/groups/dt1francoqc)  [dt1francoqc](https://www.facebook.com/groups/dt1francoqc) | - Groupe d'entraide virtuel | Personnes avec diabète de type 1 et leurs proches | Tout Québec |
| **Répertoire « Santé mentale + Diabète »**  [www.repertoire.frdj.ca](http://www.repertoire.frdj.ca/) | - Répertoire des fournisseurs de soins en santé mentale ayant suivi une formation supplémentaire en diabète | Personnes diabétiques et professionnel∙le∙s de  la santé | Tout Québec |


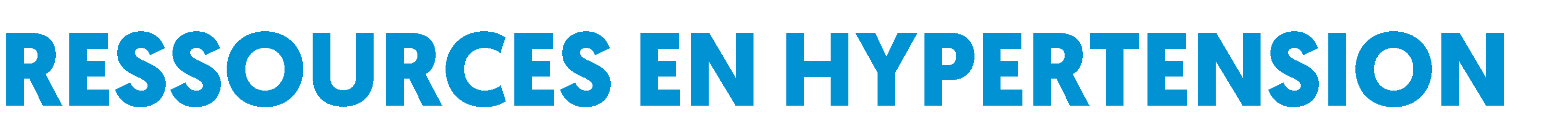


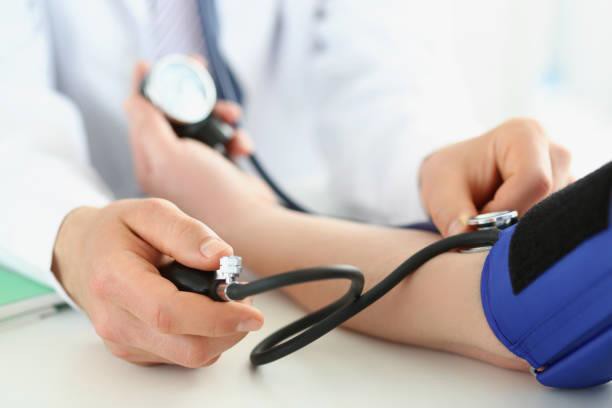


| **Nom et coordonnées** | **Description** | **Population ciblée** | **Région** |
| --- | --- | --- | --- |
| **Hypertension Canada**  [www.hypertension.ca](http://www.hypertension.ca/) | - Information utile sur le dépistage et la gestion de l’hypertension | Professionnel∙le∙s de la santé et grand  public | Tout Québec |
| **L’association Un Cœur Pour Tous**  450 760-3039  [uncoeurpourtous@hotmail.com](mailto:uncoeurpourtous@hotmail.com) | - Renseignements relatifs aux maladies cardiaques et à leurs retombées sur le plan physique et psychologique - Activités physiques personnalisées: natation, yoga sur chaise et conditionnement physique.   • Activités sociales | Personnes atteintes de haute pression et leurs proches | Lanaudière |
| **La société québécoise d'hypertension artérielle**  [www.sqha2.hypertension.qc.ca](http://www.sqha2.hypertension.qc.ca/) | - Documentation utile - Guides alimentaires - Mesure de pression à domicile | Personnes vivant avec de l'hypertension artérielle et professionnel∙le∙s de  la santé | Tout Québec |


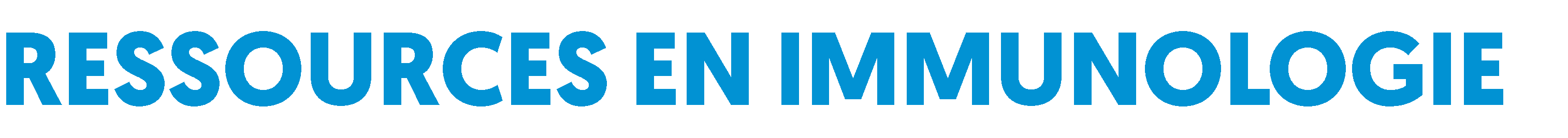


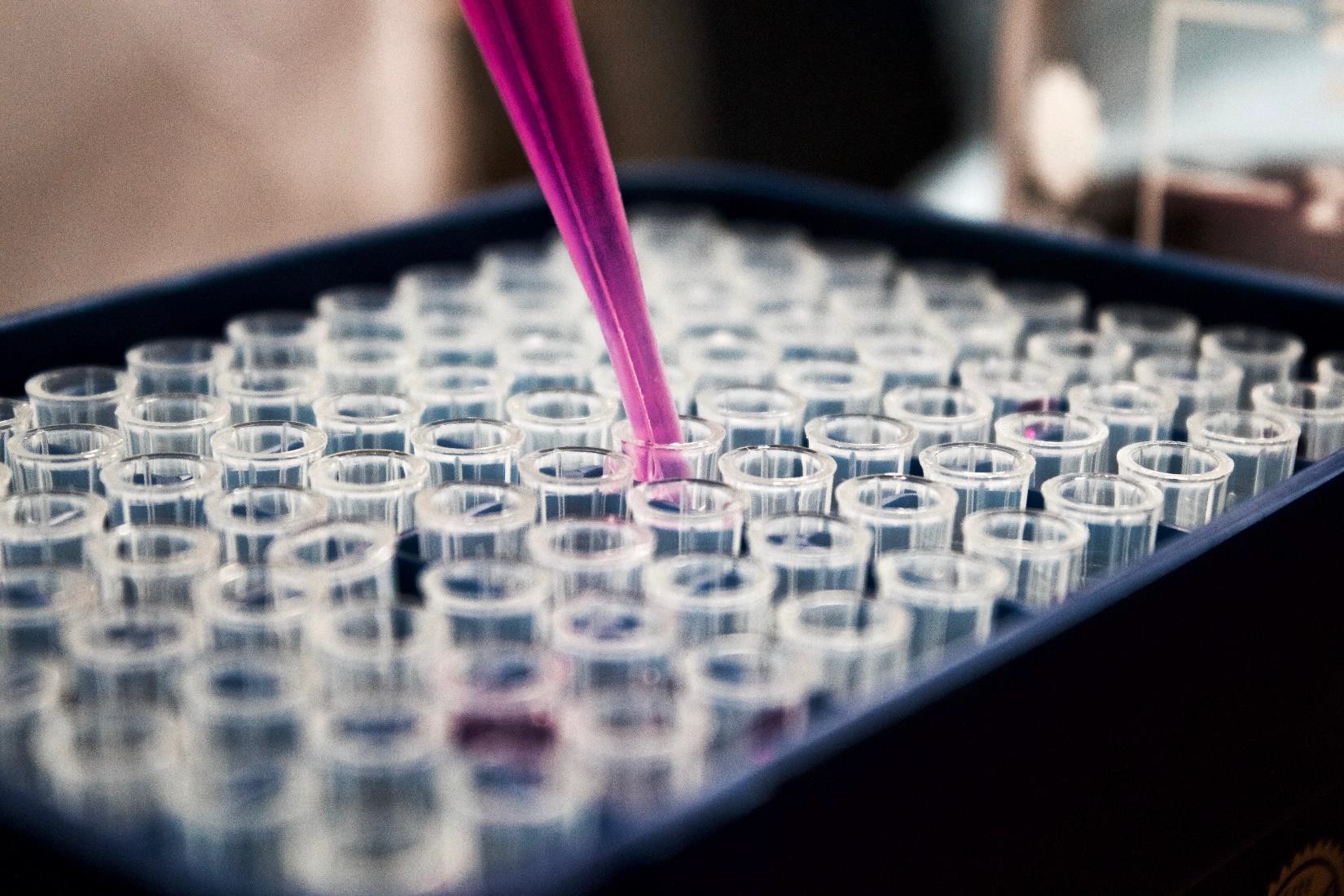


| **Nom et coordonnées** | **Description** | **Population ciblée** | **Région** |
| --- | --- | --- | --- |
| **Association de patients Déficits Immunitaires Primitifs (DIP)**  [www.associationiris.org](http://www.associationiris.org/) | - Information sur les DIP - Conseils pour mieux vivre avec les DIP - Information sur la transmission et le diagnostic - Conseil génétique | Personnes avec déficits  immunitaires primaires | Tout Québec |
| **Association des patients immunodéficients du Québec (APIQ)**  1 855 561-4563  [www.apiq.info](http://www.apiq.info/) | - Sensibilisation à l'immunodéficience - Promotion du diagnostic précoce - Soutien aux personnes touchées | Personnes avec déficits immunitaires, leurs proches et  professionnel∙le∙s de la  santé | Tout Québec |
| **Immunity Canada**  1 877-607-2476  [www.immunitycanada.org](http://www.immunitycanada.org/) | ****En anglais seulement*   - Documentation - List of resources and support - Virtual support groups | Patients with primary immunodeficiencies | Tout Québec |
| **International Patient Organisation for Primary Immunodeficiencies (IPOPI)**  [www.ipopi.org](http://www.ipopi.org/) | ****En anglais seulement*   - Leaflets for physicians and patients | Patients, relatives and physicians | Tout Québec |


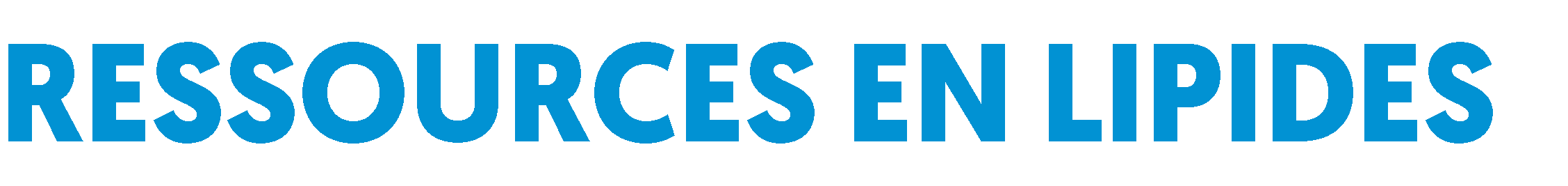


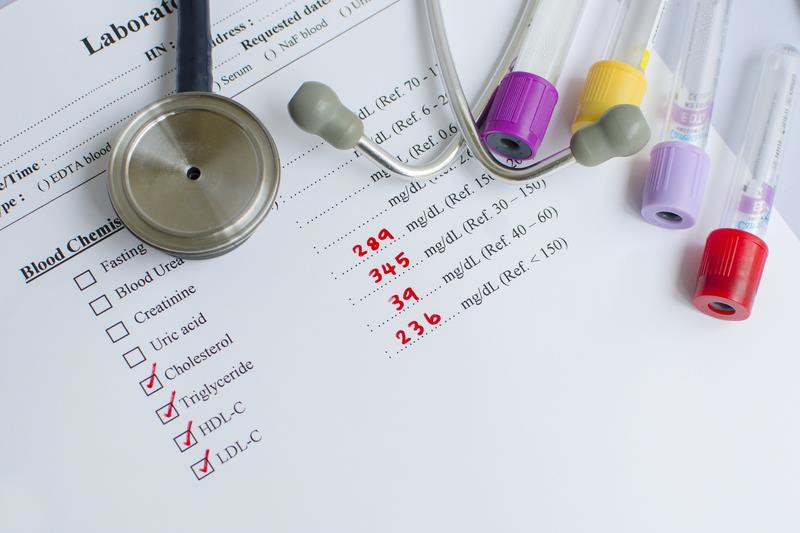


| **Nom et coordonnées** | **Description** | **Population ciblée** | **Région** |
| --- | --- | --- | --- |
| **Canadian Cardiovascular Society**  [https://ccs.ca/guideline/2021-](https://ccs.ca/guideline/2021-lipids/)  [lipids/](https://ccs.ca/guideline/2021-lipids/) | - [Documentation sur la maladie](https://ccs.ca/app/uploads/2020/11/Lipids_Gui_2016_EN.pdf) | Personnes touchées par la dyslipidémie | Tout Québec |


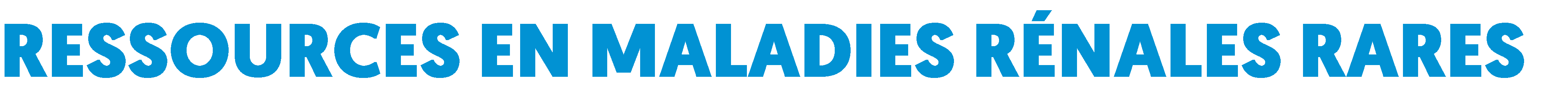


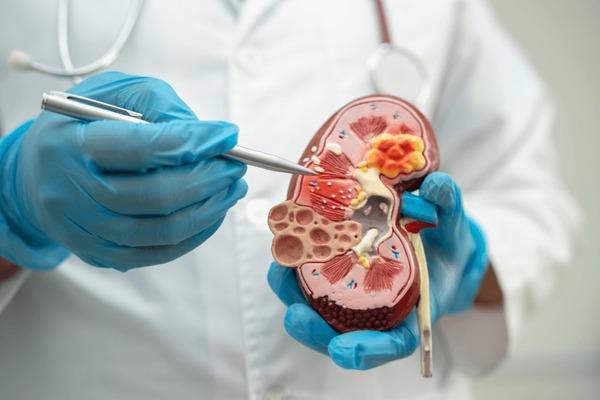


| **Nom et coordonnées** | **Description** | **Population ciblée** | **Région** |
| --- | --- | --- | --- |
| **Association générale des insuffisants rénaux (AGIR)** 514 852-9297  [www.agir.ca](http://www.agir.ca/) | - Groupes de discussion et d’entraide - Séances d’information sur l’insuffisance rénale - Ligne téléphonique d’information et de références - [Groupe de discussion virtuel](https://www.facebook.com/groups/assoagir/)   • Liste de ressources pour l’insuffisance rénale | Personnes souffrant d’insuffisance rénale et leurs proches | Tout Québec |
| **Fondation canadienne de la maladie polykystique des reins (MPR)**  1 877-410-1741  [www.finielampr.ca](http://www.finielampr.ca/) | - Recherche clinique et programme de bourse sur le sujet de la MPR - Groupes de soutien - Réseau de bénévoles | Personnes atteintes de MPR, leurs proches et bénévoles | Tout Québec |
| **Fondation canadienne du rein**  514-938-4515  [www.rein.ca](http://www.rein.ca/) | - Liste de ressources pour l’insuffisance rénale (forum virtuel, dépliants d’information, webinaires) - Programme d’aide financière à court terme - Programme de répit pour les patient∙e∙s et leurs proches | Personnes souffrant d’insuffisance rénale et leurs proches | Tout Québec |


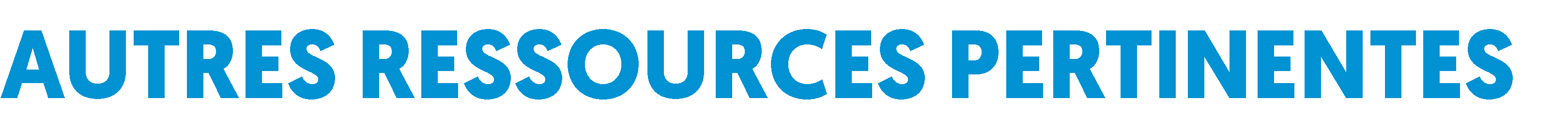


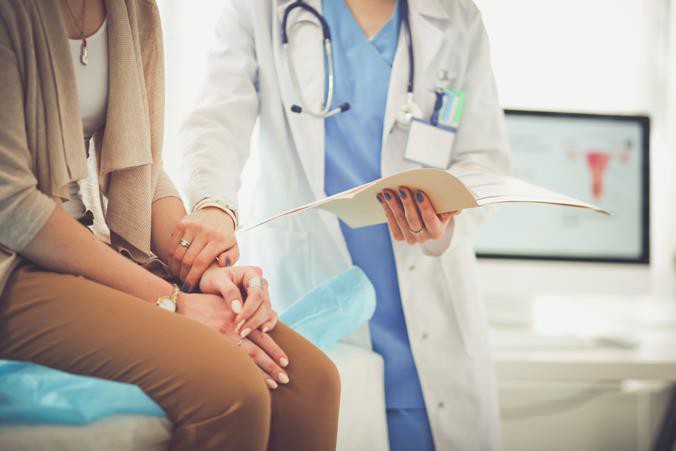


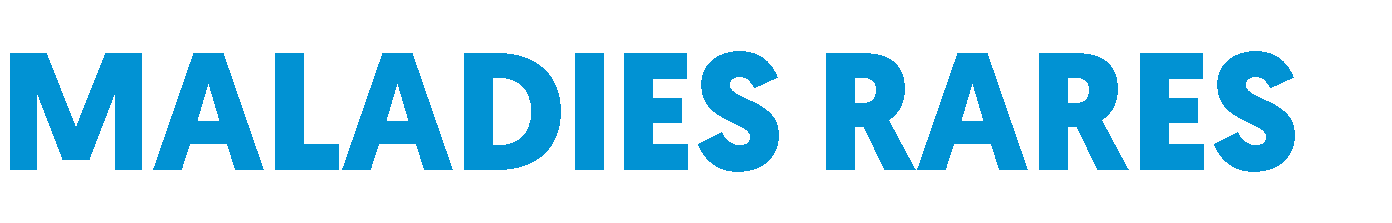


| **Nom et coordonnées** | **Description** | **Population ciblée** | **Région** |
| --- | --- | --- | --- |
| Canadian Organization for Rare Disorders (CORD)  [www.raredisorders.ca](http://www.raredisorders.ca/) | ****En anglais seulement*   - List of resources for rare disorders - Leaflets about rare disorders | People with rare disease(s) | Tout Québec |
| **Regroupement québécois des maladies orphelines (RQMO)**  1-888-822-2854  [www.rqmo.org](http://www.rqmo.org/) | - Centre IRARE: Aide pour trouver les ressources gouvernementales ou communautaires selon la maladie - Outils de recherche pour trouver des associations et groupes de soutien - Programme de jumelage | Personnes touchées par une maladie rare | Tout Québec |


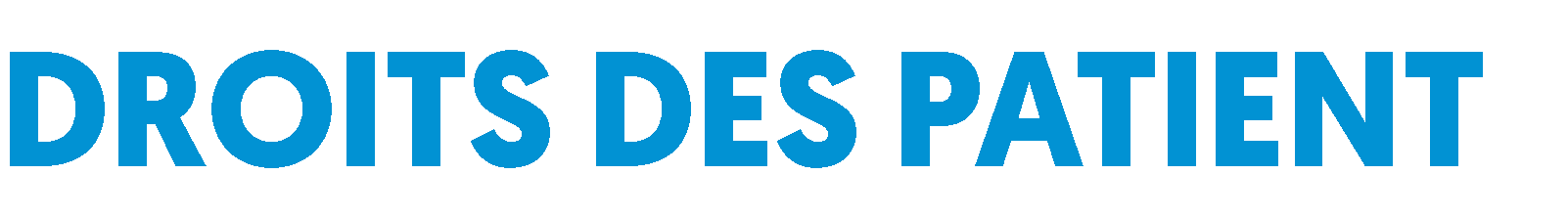

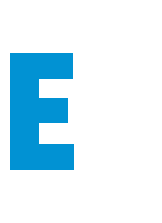

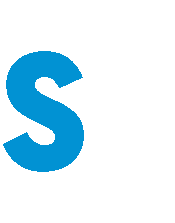


- ∙

| **Nom et coordonnées** | **Description** | **Population ciblée** | **Région** |
| --- | --- | --- | --- |
| Fédération des Centres d’assistance et d’accompagnement aux plaintes (CAAP)  1 877 767-2227  [www.fcaap.ca](http://www.fcaap.ca/) | - Information sur les droits des patient∙e∙s en vertu de la Loi sur la Santé et les Services sociaux (LSSSS) - Soutien dans les démarches - Répertoire des CAAP à travers le Québec | Patient∙e∙s, leurs proches et les professionnel∙le∙s de la santé | Tout Québec |


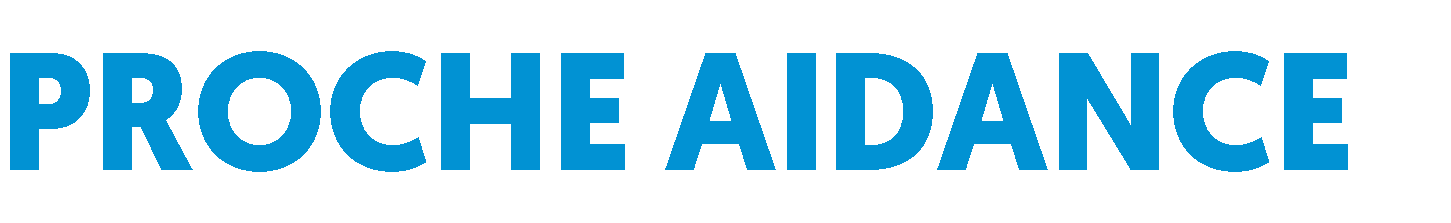


| **Nom et coordonnées** | **Description** | **Population ciblée** | **Région** |
| --- | --- | --- | --- |
| **Appui proches aidants**  1 855 852-7784  [www.lappui.org](http://www.lappui.org/) | - Ligne d’écoute - Répertoire de ressources - Formations et conseils   • | Proches aidants | Tout Québec |
| **Proche aidance Québec**  514 524-1959  <https://procheaidance.quebec/> | - Information sur la proche aidance et leurs droits - Répertoire des organismes de proche aidance dans toutes les régions du Québec | Proches aidants | Tout Québec |
